# A Randomized Phase III Study of Arfolitixorin versus Leucovorin with 5-Fluorouracil, Oxaliplatin, and Bevacizumab for First-Line Treatment of Metastatic Colorectal Cancer: The AGENT Trial

**DOI:** 10.1158/2767-9764.CRC-23-0361

**Published:** 2024-01-04

**Authors:** Josep Tabernero, Takayuki Yoshino, Sebastian Stintzing, Aimery de Gramont, Peter Gibbs, Derek J. Jonker, Peter Nygren, Christos Papadimitriou, Gerald W. Prager, Roger Tell, Heinz-Josef Lenz

**Affiliations:** 1 Vall d'Hebron Hospital Campus and Institute of Oncology (VHIO), IOB-Quiron, Barcelona, Spain.; 2 Department of Gastroenterology and Gastrointestinal Oncology, National Cancer Center Hospital East, Kashiwa, Japan.; 3 Department of Hematology, Oncology and Cancer Immunology, Charité – Universitätsmedizin Berlin, Berlin, Germany.; 4 Institute Hospitalier Franco-Britannique, Oncologie médicale, Levallois-Perret, France.; 5 Western Health – Sunshine Hospital, Medical Oncology, St. Albans, Victoria, Australia.; 6 Ottawa Hospital Research Institute, University of Ottawa, Ottawa, Canada.; 7 Department of Immunology, Genetics and Pathology, Uppsala University, Uppsala, Sweden.; 8 Oncology Unit, “Aretaieion” University Hospital, National and Kapodistrian University of Athens, Athens, Greece.; 9 Medizinische Universität Wien, Vienna, Austria.; 10 Isofol Medical AB, Gothenburg, Sweden.; 11 Division of Medical Oncology and Colorectal Cancer, Keck School of Medicine, University of Southern California, Los Angeles, California.

## Abstract

**Purpose:**

Suboptimal treatment outcomes with 5-fluorouracil (5-FU)/folate, the standard of care for metastatic colorectal cancer (mCRC), have generated interest in optimizing the folate. Arfolitixorin ([6R]-5,10-methylene-tetrahydrofolate) is an immediately active folate and may improve outcomes over the existing standard of care (leucovorin).

**Experimental Design:**

AGENT was a randomized, phase III study (NCT03750786). Patients with mCRC were randomized to arfolitixorin (120 mg/m^2^ given as two intravenous bolus doses of 60 mg/m^2^) or leucovorin (400 mg/m^2^ given as a single intravenous infusion) plus 5-FU, oxaliplatin, and bevacizumab. Assessments were performed every 8 weeks. The primary endpoint was the superiority of arfolitixorin for overall response rate (ORR).

**Results:**

Between February 2019 and April 2021, 490 patients were randomized (245 to each arm). After a median follow-up of 266 days, the primary endpoint of superiority for ORR was not achieved (48.2% for arfolitixorin vs. 49.4% for leucovorin, *P*_superiority_ = 0.57). Outcomes were not achieved for median progression-free survival (PFS; 12.8 and 11.6 months, *P* = 0.38), median duration of response (12.2 and 12.9 months, *P* = 0.40), and median overall survival (23.8 and 28.0 months, *P* = 0.78). The proportion of patients with an adverse event of grade ≥3 severity was similar between arms (68.7% and 67.2%, respectively), as was quality of life. *BRAF* mutations and *MTHFD2* expression were both associated with a lower PFS with arfolitixorin.

**Conclusions:**

The study failed to demonstrate clinical benefit of arfolitixorin (120 mg/m^2^) over leucovorin. However, it provides some useful insights from the first-line treatment setting, including the effect of gene expression on outcomes.

**Significance:**

This phase III study compared arfolitixorin, a direct-acting folate, with leucovorin in FOLFOX plus bevacizumab in mCRC. Arfolitixorin (120 mg/m^2^) did not improve the ORR, potentially indicating a suboptimal dose.

## Introduction

Globally, colorectal cancer is the third leading cancer in terms of incidence and the second leading cause of cancer-related mortality after lung cancer (1). Overall, 20% of patients with colorectal cancer have metastatic disease (mCRC) at diagnosis, and an additional 25% with initially localized disease will develop metastases (2–4). Advances in mCRC treatment over the last few decades have improved survival rates, but the prognosis is still suboptimal, with a 5-year overall survival (OS) rate of approximately 10% (5).

The combination of 5-fluorouracil (5-FU)-folate, given with oxaliplatin or irinotecan (FOLFOX or FOLFIRI) with or without bevacizumab, is a standard cytotoxic treatment for non-deficient mismatch repair/non–microsatellite instability-high mCRC (6). 5-FU, a fluoropyrimidine, functions by disrupting DNA and RNA synthesis through its metabolite [5-fluoro-2′-deoxyuridine-5-monophosphate (5-FdUMP)] (7). 5-FdUMP inhibits DNA replication directly through misincorporation into DNA, and indirectly via the formation of an inhibitory ternary complex with the enzyme thymidylate synthase (TS) and [6R]-5,10-methylene-tetrahydrofolate ([6R]-5,10-MTHF), inhibiting thymine biosynthesis and arresting DNA synthesis (7, 8).

Several studies have investigated the potential to improve outcomes in mCRC by adding additional agents to 5-FU/folate, with variable results (9, 10). Currently, treatment options for patients unresponsive to the standard-of-care options are limited. This is largely due to the lack of a treatment personalization in mCRC, which has been hindered by a limited understanding of the role of genetic factors and predictive biomarkers of response.

Folates are essential for stabilizing the enzymatic ternary complex that inhibits DNA synthesis in patients treated with 5-FU, but only limited research has explored the potential for treatment optimization by modifying the folate agent (11). Currently available folates require metabolic activation. This is a multistep process, of which the efficiency is determined by the expression of genes that encode folate transporters and folate metabolizing enzymes, and influence intratumoral folate concentrations (12, 13). On this basis, it is reasonable to hypothesize that the administration of a biologically active folate will optimize treatment outcomes in patients with mCRC, particularly patients with low folate pathway gene expression.

Arfolitixorin is the hemisulfate salt of the bioactive cofactor [6R]-5,10-MTHF (Data on file). This active thymidylate synthase cosubstrate potentiates the effect of 5-FU (8). Arfolitixorin is the only folate that is capable of directly forming an inhibitory ternary complex with the target enzyme TS and the 5-FU anabolic metabolite 5-FdUMP. This differs to currently approved folates, such as leucovorin ([6R,S]-5-formyl-tetrahydrofolate), which need to be metabolically activated to [6R]-5,10-MTHF (8). A phase I/II clinical study indicated that arfolitixorin is well tolerated in mCRC (14). This phase III study compared the clinical effectiveness of arfolitixorin versus leucovorin as part of a 5-FU/folate combination treatment in mCRC. It was the first randomized trial to examine the effect of gene expression on clinical outcomes with these agents.

## Materials and Methods

### Study Design and Participants

This multicenter, randomized, parallel-group, phase III study (NCT03750786) was conducted at 94 sites across 10 countries (Australia, Austria, Canada, France, Germany, Greece, Japan, Spain, Sweden, and the United States).

Eligible patients were ages ≥18 years at the date of informed consent and had previously untreated, biopsy-confirmed, non-resectable metastatic adenocarcinoma colorectal cancer (defined as at least one measurable lesion of metastatic disease ≥10 mm in longest diameter on axial image on CT scan, or alternatively MRI, with <5 mm reconstruction interval, or lymph node ≥15 mm in shortest axis when assessed by CT, obtained within 28 days of randomization). Patients had to have a life expectancy of >4 months, an Eastern Cooperative Oncology Group (ECOG) performance status of 0 or 1 at screening, and adequate hematologic, renal, and hepatic function (see Supplementary Table S1 for detailed eligibility criteria). The protocol was approved by the research ethics committees at each site, all patients provided written informed consent, and the trial was conducted in accordance with the Declaration of Helsinki (2008), Good Clinical Practice, and applicable regulatory and local guidelines.

### Treatments

Participants were randomized 1:1 to either arfolitixorin (the investigational arm) or leucovorin (the control arm). Randomization was stratified for geographical region (Europe vs. North America vs. Australia vs. Japan), location of primary tumor (left colon vs. right colon vs. rectum), and previous neoadjuvant/adjuvant treatment for colorectal cancer (yes vs. no). All participants received racemic leucovorin, which was provided to all sites by the study sponsor. Both arms were treated at the same dose intensity with sequential bevacizumab 5 mg/kg intravenous infusion, oxaliplatin 85 mg/m^2^ intravenous infusion, and 5-FU (which included a 400 mg/m^2^ intravenous bolus over 2−4 minutes and a separate 2,400 mg/m^2^ continuous intravenous infusion over 46 hours). On the basis of the results of the previous phase I/II study of arfolitixorin, in which a calculated dose of 120 mg/m^2^ (given as two intravenous bolus injections 30 minutes apart) was selected as the dose for further investigation (14), the investigational arm received two rapid intravenous bolus doses of arfolitixorin 60 mg/m^2^. The first was administered 30 ± 5 minutes after the 5-FU bolus dose followed by another 30–60 minutes after the first (concomitantly with the continuous infusion of 5-FU). The control arm received racemic leucovorin 400 mg/m^2^ as a single intravenous infusion prior to the intravenous bolus dose of 5-FU. The calculation of body surface area to determine the dose of arfolitixorin, leucovorin, 5-FU, and oxaliplatin was based on the DuBois formula (15). The dose of bevacizumab was based on body weight, as per the manufacturer's instruction.

The treatment cycle started within 3 days of randomization and was repeated every 14 days (with a maximum duration of 21 days) until progressive disease (PD), unacceptable toxicity, or other reason warranting discontinuation. Assessment visits were performed every 8 weeks from baseline and could be performed up to 7 days after the 8-week timepoint. At each treatment visit, the collection of blood, urine samples, and the recording of vital signs were done before the start of treatment.

### Study Endpoints

All efficacy endpoints were assessed by blinded independent central review (BICR) using RECIST 1.1 and were based on the presence of PD on CT/MRI scans of the thorax, abdomen, and pelvis. The primary endpoint was overall response rate (ORR), chosen to meet regulatory requirements, and defined as the best overall response (BOR) recorded from the start of the study treatment until the end of treatment, or the last available assessment at the time of database lock. This was subsequently confirmed 8 weeks after its onset. The secondary endpoints were progression-free survival (PFS; the time from randomization to the first occurrence of tumor progression or death), duration of response (DoR; the time from when ORR was achieved until PD was first objectively documented), OS (also included as a safety endpoint to meet regulatory requirements, and evaluate whether arfolitixorin can be considered non-detrimental in comparison with leucovorin), quality of life [assessed using the EQ-VAS instrument (16)], safety and tolerability, and the ability to undergo surgical resection. Safety was assessed on the basis of the frequency and severity of adverse events (AE) and serious adverse events (SAE), including clinically significant laboratory abnormalities, using NCI Common Terminology Criteria for Adverse Events, version 5.0.

Exploratory endpoints included analysis of folate pathway gene expression, and the outcome of clinical endpoints including BOR, PFS, and recurrence-free survival (RFS) in patients undergoing resection of metastases (defined as the time between the first surgery with complete removal of the metastasis and recurrence of the disease or death from any cause). Gene expression analysis was performed on tumor biopsy samples taken from consenting patients at baseline using qPCR to measure expression levels of genes associated with mCRC (Supplementary Table S2). Gene expression levels were quantified using real-time PCR and correlated with treatment outcomes.

### Statistical Analysis

A minimum of 440 patients were needed to give an 80% power for detecting a 13.5% difference on a two-sided test of the superiority of arfolitixorin over leucovorin. The conditional power for both ORR and PFS was calculated at the interim efficacy analysis [which was performed by the Data and Safety Monitoring Board (DSMB)] when the 16-week BICR evaluation had been performed for the 330th patient). The outcome determined whether enrollment continued as planned or was expanded by 50% to accrue 660 patients.

Efficacy analyses were performed using the intent-to-treat (ITT) population (comprising all randomized patients). The primary endpoint of ORR was also evaluated in the per protocol population (comprising all patients who completed the study in accordance with the protocol). The primary endpoint of ORR was summarized on the basis of proportions, and a stratified Cochran Mantel–Haenszel *χ*^2^ test was used to evaluate differences between treatment arms. PFS, DOR, and RFS were estimated using the Kaplan–Meier approach, which was used to obtain point estimates and confidence intervals (CI). Safety endpoints were analyzed in the safety analysis set (comprising all patients who received ≥1 dose of study drug and had at least one postbaseline safety assessment). The proportion of patients in each arm undergoing surgical resection was evaluated using Fisher exact test.

Statistical significance was accepted on the basis of a two-tailed *P* value of ≤0.05. All statistical analyses for efficacy and safety endpoints were conducted using SAS version 9.4 or higher. Gene expression analyses were performed without imputations using R statistical software. Data evaluation was conducted by investigators of participating sites and the sponsor.

### Data Availability Statement

These data are available at ClinicalTrials.gov.

## Results

### Trial and Patient Characteristics

**FIGURE 1:**
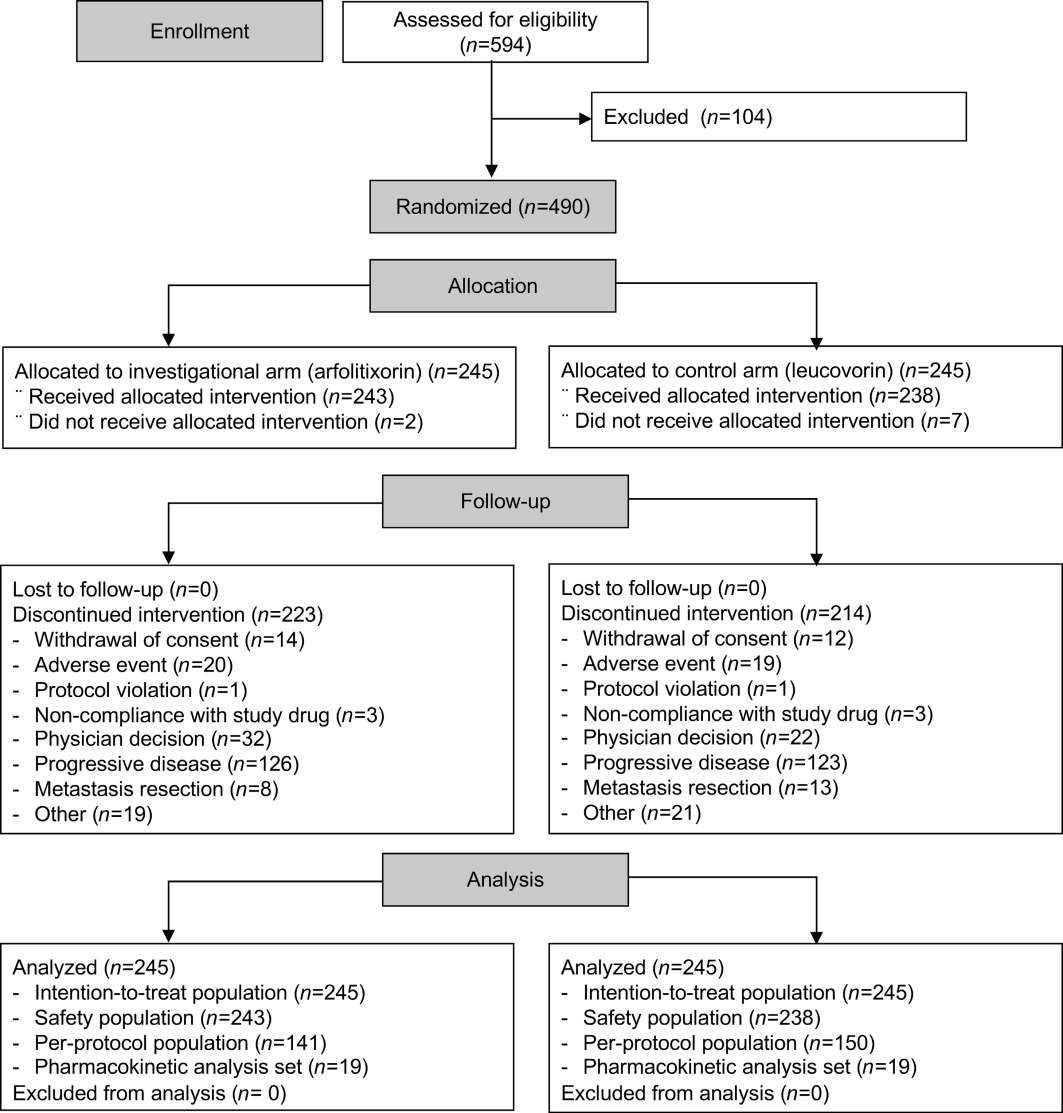
Patient disposition.

Between December 2018 and April 2021, 594 patients were screened, 490 patients were randomized, and 245 were assigned to each treatment arm (forming the ITT population; Fig. 1). This included 58 patients from Japan, who were enrolled to meet the request of the Japanese regulatory authorities to enroll approximately 12.7% of the total study population from Japan. The interim efficacy analysis was performed using a data cutoff of January 12, 2021. Following DSMB review, the sample size was not increased. The enrolled population was strongly representative of the real-world population with colorectal cancer (Supplementary Table S3).

Baseline characteristics were generally well balanced between treatment arms (Table 1). The mean age of patients was 62.4 years in the arfolitixorin arm and 62.6 years in the leucovorin arm, most patients were male (66.1% and 61.6%, respectively), the median time since diagnosis of metastasis was 1.18 and 1.25 months respectively, and the majority of patients who were tested for *KRAS* mutations expressed mutant *KRAS* (62.3% and 65.5%, respectively).

**TABLE 1:** Baseline patient demographics and characteristics

Characteristic	Arfolitixorin arm (*n* = 245)	Leucovorin arm (*n* = 245)
**Sex, *n* (%)**		
Male	162 (66.1)	151 (61.6)
Female	83 (33.9)	94 (38.4)
**Median age, years (IQR)**	63 (56.0–71.0)	64 (55.0–71.0)
**Race, *n* (%)**		
White	179 (73.1)	192 (78.4)
Asian	36 (14.7)	35 (14.3)
Other	11 (4.5)	9 (3.7)
Missing	19 (7.8)	9 (3.7)
**Time since initial diagnosis, months**		
Mean ± SD	9.73 ± 20.887	7.7 ± 15.151
Median (IQR)	1.58 (1.08–4.70)	1.68 (0.99–3.48)
**Time since diagnosis of metastasis, months**		
Mean ± SD	2.66 ± 6.272	2.58 ± 5.537
Median (IQR)	1.18 (0.89–1.77)	1.25 (0.89–1.84)
**Primary tumor location, *n* (%)**		
Right colon	86 (35.1)	82 (33.5)
Left colon	84 (34.3)	84 (34.3)
Rectum	75 (30.6)	79 (32.2)
**Primary tumor still in place, *n* (%)**	145 (59.2)	144 (58.8)
**Patients with surgical procedure, *n* (%)**	109 (44.5)	109 (44.5)
**Metastatic sites (a patient may have more than one), *n* (%)**		
Liver	177 (72.2)	184 (75.1)
Lung	110 (44.9)	119 (48.6)
Other	118 (48.2)	94 (38.4)
**ECOG score, *n* (%)**		
0	163 (67.0)	156 (63.7)
1	79 (32.2)	82 (33.5)
2	1 (0.4)	0
Not available	2 (0.8)	7 (2.9)
**Gene mutation testing performed, *n* (%)**	187 (76.3)	186 (75.9)
***BRAF*, *n* (%)**	109 (44.5)	131 (53.5)
Wild-type	91 (83.5)	118 (90.1)
Mutant	18 (16.5)	13 (9.9)
***KRAS*, *n* (%)**	162 (66.1)	171 (69.8)
Wild-type	61 (37.7)	59 (34.5)
Mutant	101 (62.3)	112 (65.5)
***NRAS*, *n* (%)**	101 (41.2)	128 (52.2)
Wild-type	85 (84.2)	114 (89.1)
Mutant	16 (15.8)	14 (10.9)
**Other gene mutation tested**	78 (31.8)	75 (30.6)

Abbreviations: ECOG, Eastern Cooperative Oncology Group; IQR, interquartile range; SD, standard deviation.

At the time of analysis, 44 patients in the safety population remained on treatment and 437 had discontinued. The most common reason for treatment discontinuation was PD, which accounted for discontinuation in 126 (51.9%) patients in the arfolitixorin arm and 123 (51.7%) patients in the leucovorin arm (Supplementary Table S4). The per protocol population consisted of 141 and 150 patients (57.6% and 61.2% of the ITT population) in the arfolitixorin and leucovorin arms, respectively. The difference was due to the high number of protocol deviations. The most common deviation was an incorrect or missing procedure or assessment, which affected 42 (17.1%) and 48 (19.6%) patients, respectively. A COVID-19–related major protocol deviation occurred in 4 patients (1.6%) and 1 patient (0.4%), respectively.

### Interim and Primary Efficacy Results

At the time of the interim analysis, the ORR was 48.0% (95% CI: 40.3−55.6) for arfolitixorin and 43.4% (95% CI: 35.7−51.2) for leucovorin [OR = 0.78 (95% CI: 0.20−3.00); *P* = 0.72] (Supplementary Table S5). Median PFS was 11.1 months for arfolitixorin (95% CI: 9.2−12.3) and 11.0 months for leucovorin (95% CI: 9.0−12.0) [HR = 1.00 (95% CI: 0.71−1.41); *P* = 0.98].

The data cutoff for the primary efficacy analysis was April 10, 2022. At this timepoint, the median follow-up was 266 days. The primary endpoint of ORR did not achieve superiority [48.2% (95% CI: 41.8−54.6) for arfolitixorin and 49.4% (95% CI: 43.0−55.8) for leucovorin; *P* = 0.57] (Table 2). Analyses in the per protocol population (*n* = 141 for arfolitixorin and 150 for leucovorin) demonstrated the same trend for ORR [44.0% (95% CI: 35.6−52.6) for arfolitixorin and 50.0% (95% CI: 41.7−58.3) for leucovorin].

**TABLE 2:** Primary efficacy endpoint: ORR (by BICR in ITT population)

Confirmed best overall response, *n* (%)	Arfolitixorin arm (*n* = 245)	Leucovorin arm (*n* = 245)
CR	2 (0.8)	5 (2.0)
PR	116 (47.3)	116 (47.3)
SD	106 (43.3)	86 (35.1)
PD	7 (2.9)	11 (4.5)
ORR (CR + PR)	118 (48.2)	121 (49.4)
DCR (CR + PR + SD)	224 (91.4)	207 (84.5)
Not evaluable, no BOR available, or non-CR/non-PD^a^	14 (5.6)	27 (10.9)

Abbreviations: BOR, best overall response; CR, complete response; DCR, disease control rate; ITT, intent-to-treat; ORR, overall response rate; PD, progressive disease; PR, partial response; SD, stable disease.

^a^Non-CR/non-PD refers to patients with no target lesions, only non-target lesion classified as non-CR/non-PD as best overall response.

### Secondary Efficacy and Quality-of-life Results

Median PFS after censoring was not significantly different between treatment arms [12.8 months for arfolitixorin and 11.6 months for leucovorin, HR = 0.96 (95% CI: 0.74−1.25); *P* = 0.38] (Supplementary Fig. S1; Supplementary Table S6). Median DoR after censoring, measured in patients who achieved at least a partial treatment response (*n* = 118 for arfolitixorin and 121 for leucovorin), demonstrated the same trend [12.2 and 12.9 months, respectively, HR = 0.95 (95% CI: 0.64–1.41); *P* = 0.40] (Supplementary Fig. S2; Supplementary Table S7). Similarity between treatment arms was also observed for OS [23.8 months for arfolitixorin and 28.0 months for leucovorin, HR = 1.114 (95% CI: 0.85−1.47); *P* = 0.78]. The 6-month OS rate was 96.3% for arfolitixorin and 94.4% for leucovorin. Corresponding values were 82.5% and 80.7% at 12 months, 66.0% and 69.4% at 18 months, and 49.7% and 55.8% at 24 months, respectively.

The proportion of patients in each arm undergoing surgical resections of either the primary tumor, a metastatic tumor site, or both, was not significantly different for curative intent surgery (Fisher exact *P* = 0.79) or palliative intent surgery (Fisher exact *P* = 0.64).

Quality of life, measured using EQ-VAS patient-reported outcomes, was comparable between groups at the end of treatment (the mean score was 69.6 ± 20.42 for arfolitixorin and 73.1 ± 19.34 for leucovorin; Supplementary Table S8; Supplementary Fig. S3).

### Safety and Tolerability Results

Safety analyses were conducted in 481 treated patients (243 in the arfolitixorin arm and 238 in the leucovorin arm), which constituted the safety analysis set. The overall incidence of AEs was similar in both treatment arms. Nearly all patients had ≥1 AE (241 patients in the arfolitixorin arm and 236 patients in the leucovorin arm, equating to 99.2% of patients in each arm). Patients with at least one drug-related AE accounted for less than half of these (46.9% of patients in the arfolitixorin arm and 43.3% of patients in the leucovorin arm; Supplementary Table S9).

The proportion of patients with an AE of grade ≥3 severity was similar between arms (68.7% in the arfolitixorin arm and 67.2% in the leucovorin arm), as was the proportion of patients with AEs of grade ≥3 severity that were positively attributed to the study treatment (14.8% vs. 10.9%; Supplementary Table S10).

The distribution of AEs was generally comparable between arms. The most common categories of AEs affecting ≥10% of patients in both were gastrointestinal disorders (affecting 81.9% patients on arfolitixorin and 81.1% patients on leucovorin), nervous system disorders (affecting 82.3% and 79.4%, respectively), general/administration site disorders (affecting 72.4% and 68.9%, respectively), and blood/lymphatic system disorders (affecting 41.2% and 42.4%, respectively; Table 3). Neurotoxicity was the only AE for which there appeared to be a substantial difference between arfolitixorin and leucovorin arms (16.5% vs. 9.2%), but the significance was not evaluated, and this difference was not considered causally related to the study treatment.

**TABLE 3:** AEs with frequency ≥10% in either trial arm (safety population)

System organ class and preferred term	Arfolitixorin arm (*N* = 243)	Leucovorin arm (*N* = 238)
**All system organ classes, *n* (%)**		
Patients with at least one AE	239 (98.4)	231 (97.1)
Total number of AEs	1,523	1,514
**Gastrointestinal disorders, *n* (%)**		
Patients with at least one AE	199 (81.9)	193 (81.1)
Nausea	117 (48.1)	123 (51.7)
Diarrhea	118 (48.6)	113 (47.5)
Constipation	69 (28.4)	64 (26.9)
Vomiting	60 (24.7)	55 (23.1)
Stomatitis	52 (21.4)	62 (26.1)
Abdominal pain	51 (21.0)	50 (21.0)
Total number of AEs	467	467
**Nervous system disorders, *n* (%)**		
Patients with at least one AE	200 (82.3)	189 (79.4)
Peripheral sensory neuropathy	66 (27.2)	72 (30.3)
Neuropathy peripheral	73 (30.0)	64 (26.9)
Paresthesia	36 (14.8)	42 (17.6)
Dysgeusia	40 (16.5)	37 (15.5)
Neurotoxicity	40 (16.5)	22 (9.2)
Dysesthesia	26 (10.7)	31 (13.0)
Headache	25 (10.3)	24 (10.1)
Total number of AEs	306	292
**General disorders and administration site conditions, *n* (%)**		
Patients with at least one AE	176 (72.4)	164 (68.9)
Fatigue	108 (44.4)	103 (43.3)
Mucosal inflammation	53 (21.8)	49 (20.6)
Asthenia	48 (19.8)	42 (17.6)
Pyrexia	45 (18.5)	39 (16.4)
Total number of AEs	254	233
**Blood and lymphatic system disorders, *n* (%)**		
Patients with at least one AE	100 (41.2)	101 (42.4)
Neutropenia	60 (24.7)	51 (21.4)
Anemia	44 (18.1)	51 (21.4)
Thrombocytopenia	32 (13.2)	28 (11.8)
Total number of AEs	136	130
**Investigations, *n* (%)**		
Patients with at least one AE	70 (28.8)	72 (30.3)
Neutrophil count decreased	53 (21.8)	62 (26.1)
Platelet count decreased	33 (13.6)	35 (14.7)
White blood cell count decreased	24 (9.9)	25 (10.5)
Total number of AEs	110	122
**Metabolism and nutrition disorders, *n* (%)**		
Patients with at least one AE	66 (27.2)	60 (25.2)
Decreased appetite	66 (27.2)	60 (25.2)
Total number of AEs	66	60
**Respiratory, thoracic, and mediastinal disorders, *n* (%)**		
Patients with at least one AE	64 (26.3)	62 (26.1)
Epistaxis	64 (26.3)	62 (26.1)
Total number of AEs	64	62
**Vascular disorders, *n* (%)**		
Patients with at least one AE	55 (22.6)	60 (25.2)
Hypertension	55 (22.6)	60 (25.2)
Total number of AEs	55	60
**Skin and subcutaneous tissue disorders, *n* (%)**		
Patients with at least one AE	40 (16.5)	55 (23.1)
Alopecia	21 (8.6)	32 (13.4)
Palmar-plantar erythrodysesthesia syndrome	20 (8.2)	29 (12.2)
Total number of most common AEs	41	61
**Infections and infestations, *n* (%)**		
Patients with at least one AE	24 (9.9)	27 (11.3)
Urinary tract infection	24 (9.9)	27 (11.3)
Total number of AEs	24	27

Abbreviation: AE, adverse event.

SAEs affected 81 patients (33.3%) in the arfolitixorin arm and 86 patients (36.1%) in the leucovorin arm (Supplementary Table S11). The proportion of patients with SAEs that were considered related to either arfolitixorin or leucovorin was very low and similar between arms (3.3% in the arfolitixorin arm and 3.4% in the leucovorin arm). The most common SAE in both arms was gastrointestinal disorders, which affected 36 patients in the arfolitixorin arm (14.8%) and 35 patients in the leucovorin arm (14.7%).

AEs of special interest (AESI) occurred in 180 patients (74.1%) in the arfolitixorin arm and 171 (71.8%) patients in the leucovorin arm. The number of events relating to AESIs was also similar in both arms (283 vs. 275 events, respectively). Diarrhea was the most common AESI, affecting 48.6% and 47.5% of patients, respectively. Most cases were of grade 1 severity (Supplementary Table S12).

Overall, 219 deaths were reported [119 in the arfolitixorin arm (49.0% of participants) and 100 in the leucovorin arm (42.0% of participants)]. Most deaths were due to PD, which accounted for deaths in 103 (42.0%) patients in the arfolitixorin arm and 79 (32.2%) patients in the leucovorin arm. The proportion of deaths attributable to PD was numerically higher in the arfolitixorin arm (formal statistical comparison was not performed). Furthermore, 205 deaths (93.6%) occurred in the follow-up or after 30 days from study drug discontinuation, indicating that most deaths are treatment-unrelated. Fatal AEs were experienced in 8 patients in each arm (3.3% of patients in the arfolitixorin arm and 3.4% in the leucovorin arm, respectively). Only 2 of these patients had COVID-19 listed as a cause of death, and were both in the arfolitixorin arm.

### Exploratory Analyses

The median RFS was numerically higher for arfolitixorin, but the difference did not reach statistical significance [10.6 months for arfolitixorin and 5.6 months for leucovorin, HR = 0.97 (95% CI: 0.28−3.31); one-sided *P* = 0.48] (Supplementary Table S13; Supplementary Fig. S4).

**FIGURE 2:**
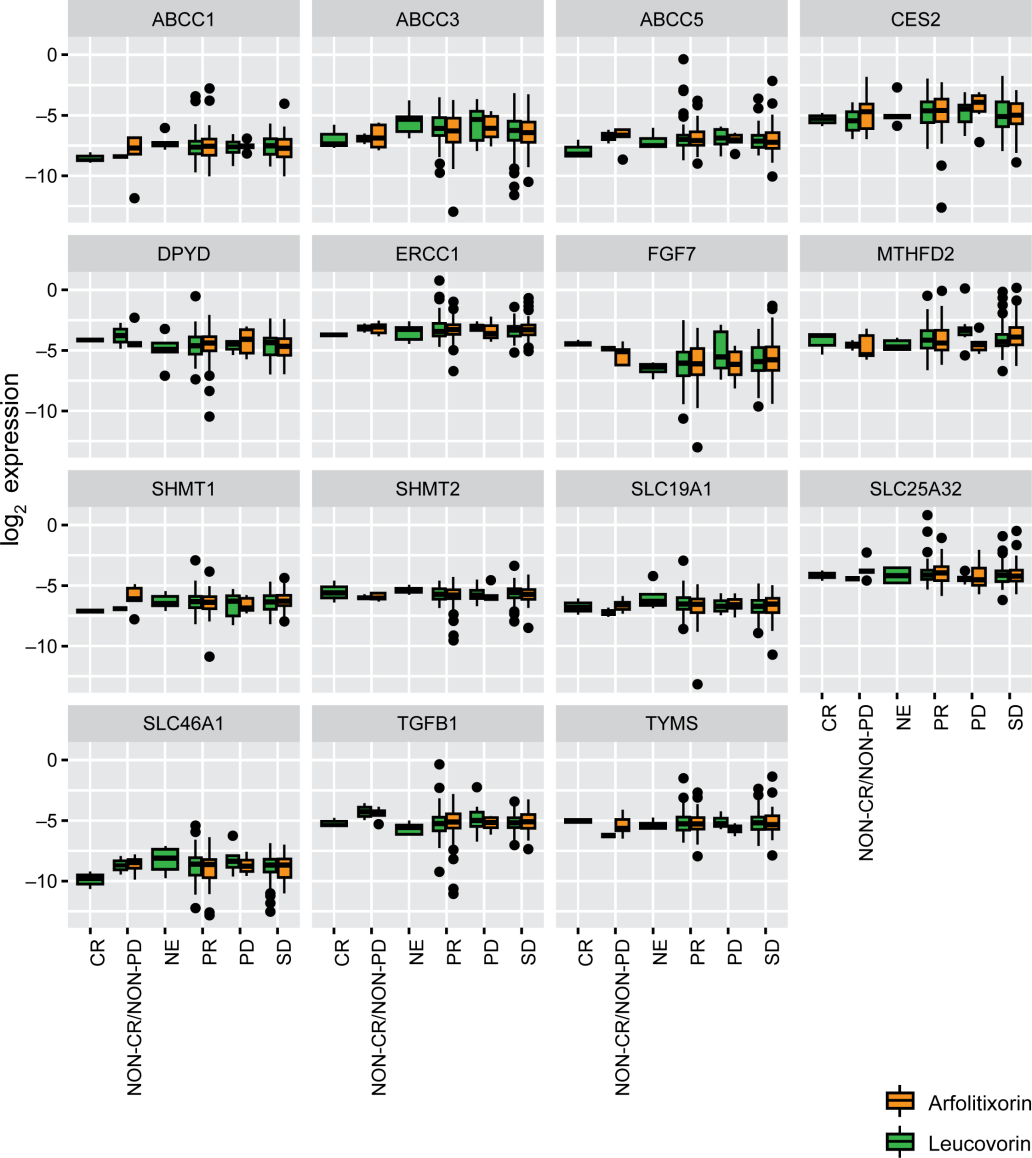
Gene expression across responses categories and treatments. Dots represent outliers. CR, complete response; NE, non-evaluable; PR, partial response; PD, progressive disease; SD, stable disease.

Gene expression analyses were conducted in tumoral biopsy samples obtained from 221 patients in the arfolitixorin arm and 211 patients in the leucovorin arm, for which the most common treatment response was a partial response (PR; 48.9% and 49.8%, respectively). Mutations in *BRAF* were associated with significantly lower PFS in the arfolitixorin arm but not in the leucovorin arm (*P* = 0.02; Supplementary Fig. S5). High *ERCC1* expression was associated with a lower median PFS and a poorer PFS prognosis in the leucovorin arm but not in the arfolitixorin arm (Supplementary Fig. S6). High *MTHFD2* expression was associated with a lower median PFS and poorer PFS prognosis independent of treatment assignment (Supplementary Fig. S7). The other genes evaluated did not have a significant impact on PFS, and differences in gene expression levels across different response categories were minimal (Fig. 2).

The ORR in the Japanese subgroup (*n* = 58) was 51.7% for arfolitixorin (95% CI: 32.5−70.6) and 72.4% (95% CI: 52.8−87.3) for leucovorin [risk difference = −0.21 (95% CI: −0.45 to 0.04)]. Although this difference was nonsignificant, it was greater than the difference that was observed in the overall study population (48.2% and 49.4%, respectively). The median PFS in this subgroup was 12.9 and 14.7 months, respectively [HR = 1.37 (95% CI: 0.65−2.9); *P* = 0.66]. Other efficacy results from this subgroup are provided in Supplementary Table S14A–S14F. The proportions of patients in this subgroup with an AE of grade ≥3 severity (58.6% and 62.1%, respectively) were similar to the overall study population.

The ORR was numerically similar in both trial arms across other prespecified subgroups, including those defined according to age, gender, ECOG performance status, location of the primary tumor, and metastatic sites (Supplementary Table S15). There was a trend toward a higher ORR with arfolitixorin in the North America subgroup (60.3% vs. 50.8%) and a lower ORR with arfolitixorin in the Japanese subgroup (51.7% vs. 72.4%). The only exception was for the variable “high neutrophil/leukocyte ratio.” Patients with an abnormal, not clinically significant value (*n* = 25) had an ORR of 70.0% (95% CI: 34.7–93.3) in the arfolitixorin arm and 26.7% (95% CI: 7.7–55.1) in the leucovorin arm. Patients with normal values (*n* = 85) had ORRs of 34.3% (95% CI: 19.1–52.2) and 62.0% (95% CI: 47.1–75.3), respectively.

## Discussion

The current study, which was designed to test the superiority of arfolitixorin over leucovorin as a component of FOLFOX and bevacizumab for the primary endpoint of ORR in the first-line treatment of mCRC, failed to demonstrate superiority. However, arfolitixorin demonstrated manageable safety and tolerability. To our knowledge, this study is the only randomized phase III trial to analyze the effect of gene expression on treatment outcomes with folate in mCRC. Of the genes evaluated for treatment interactions, only mutations in *BRAF*, and high expression of *ERCC1* and *MTHFD2*, were associated with treatment outcomes. The findings add to the evidence relating to the 5-FU/folate treatment backbone, and suggest that the determinants of treatment response in mCRC are multifactorial and only partly due to genetic factors.

The reasons for the greater difference in ORR between treatment groups in the American and Japanese subgroups may reflect geographic differences in patient and tumor characteristics. U.S. data indicated a 9% increase in the proportion of colorectal cancer cases in patients younger than 55 years, and 4% increase in the proportion of left-sided tumors, between 1995 and 2019 (17). Both factors are associated with greater clinical benefit from 5-FU–based treatment (18, 19), and this may be synergistically enhanced with an active folate. In Japan, the incidence of colorectal cancer has increased due to lifestyle factors such as increased smoking (20). Smoking reduces the anticancer activity of 5-FU–based therapy (21), and, conversely, may diminish the benefit of the active folate, potentially accounting for the observed trend.

Arfolitixorin is used as a stable formulation (Data on file). Unlike clinically available folates that require metabolic conversion to active metabolites, arfolitixorin directly stabilizes 5-FdUMP to inhibit TS, which arrests DNA synthesis with optimal biochemical efficiency (8). It was previously evaluated as an adjunct to 5-FU in a phase I/II study, which evaluated its efficacy and safety alone and in combination with irinotecan or oxaliplatin (with or without bevacizumab; ref. 14). That study, which enrolled pretreated and treatment-naïve patients, reported a reduction of ≥20% in tumor size following 8 weeks of treatment in 25% of patients who were evaluated for efficacy. This indicates that substituting arfolitixorin for leucovorin may improve outcomes in these pretreated patients, and justifies the current study in which the enrolled population exclusively comprised patients with untreated advanced colorectal cancer. The choice of racemic leucovorin instead of levoleucovorin is justified on the basis of evidence indicating that levoleucovorin, compared with racemic leucovorin, does not improve efficacy or safety in gastrointestinal cancers (22).

The dose of arfolitixorin used in this study (120 mg/m^2^) was deemed adequate for efficacy based on the findings of the published phase I/II study, in which the overall disease control rate in patients in the efficacy analysis set (who received arfolitixorin 30, 60, 120, or 240 mg/m^2^) was 73.7% (14). A subanalysis of that study also indicated that plasma deoxyuridine (a surrogate marker of cytotoxicity and early clinical response) was significantly higher with increasing dose of arfolitixorin (*P* = 0.023; ref. 23). Consequently, this was the dose evaluated in this phase III study. However, these results suggest this dose may be inadequate for eliciting a treatment response. The higher dose of 240 mg/m^2^ that was evaluated in the phase I/II study may improve outcomes (although the small number of patients enrolled in that study precluded formal statistical comparison of efficacy outcomes between dosing arms).

The results of the current study indicate that arfolitixorin was not superior to leucovorin for ORR (48.2% and 49.4%, respectively; *P* = 0.57) and did not improve PFS, DoR, or OS. It was unclear why arfolitixorin did not elicit improved outcomes over leucovorin. However, as it is a direct-acting folate, use of a suboptimal dose is more likely to be the explanation than pharmacokinetic factors (although pharmacokinetic outcomes were not evaluated in this study). However, OS in this study (23.8 months for arfolitixorin and 28.0 months for leucovorin) was considered reasonable given that all patients were treated first line, and approximately two-thirds of patients expressed mutant *KRAS*.

The tolerability outcomes of this study concur with the findings of the phase I/II study, which also indicated that arfolitixorin is well tolerated (14). In this study, safety outcomes were generally comparable between treatment arms. Most deaths in both arms were due to PD, the proportion of which was numerically higher in the arfolitixorin arm (42.4% vs. 33.2% of participants). Conversely, the proportion of deaths not attributed to PD was higher in the leucovorin arm. One potential explanation is the indication of a more advanced disease state in the arfolitixorin arm postrandomization (time from initial diagnosis ranged from 0.1–150 months compared with 0.3–103.7 months in the leucovorin arm, and time from initial diagnosis of metastatic disease ranged from 0.1–60.6 months compared with 0.1–41.5 months, respectively).

This study evaluated the effect of the expression of a panel of genes on PFS, but only observed treatment effects for *BRAF*, *ERCC1*, and *MTHFD2*. *MTHFD2* is a folate cycle enzyme that is associated with purine synthesis (24), and promotes immune evasion through programmed death-ligand 1 upregulation (25). It was associated with poorer outcomes in both treatment arms, suggesting that some aspects of colorectal cancer tumor cell function may be sufficiently dysregulated to mitigate the cytotoxic effect of folate treatment. However, this explanation is speculative, and it is noteworthy that most patients from whom tumor biopsies were obtained had only achieved a PR [213 of 432 patients (49.3%) included in the gene expression analyses] or stable disease (SD; 171 of 432 patients [39.6%]), limiting the generalizability of the gene expression results.

The gene expression analyses complement existing analyses of biopsied tissue samples from colorectal tumors in non-randomized, non-interventional studies (12, 26, 27). The existing evidence indicates differential expression of folate pathway genes, including *RFC-1, FPGS, GGH,* and *TS*, in tumoral colorectal mucosa compared with normal mucosa (26). Correlations between gene expression and prognosis have indicated that mucosal expression of *FPGS* is an independent prognostic marker (26), and tumoral expression of *SLC46A1/PCFT, SLC19A1/RFC-1, ABCC3/MRP3, GGH*, and *MTHFD1 L* are associated with enhanced disease-free survival (12, 27). The lack of treatment interactions for the majority of the genes in the panel that was evaluated in this study may reflect methodologic differences. For example, the study by Odin and colleagues evaluated the effect of *SLC46A1*, *SLC19A1*, and *ABCC3* on disease-free survival (12), whereas the current study evaluated their effect on PFS, precluding like-for-like comparison.

The lack of a clear association between gene expression and treatment outcomes suggests that there are other, potentially unelucidated, determinants of the clinical response to FOLFOX plus bevacizumab, apart from the rate of folate metabolism. Further research on this topic is justified. An unmet need persists for an enhanced understanding of how other clinical biomarkers, apart from genetic biomarkers, predict treatment outcomes with 5-FU–based cytotoxic chemotherapy, to enable patient stratification based on the likelihood of treatment response.

Limitations are mainly due to differences in the therapeutic regimens between arms, which resulted in the study being deployed as an open-label trial and may have impacted the outcomes. This included differences in the color of the injection fluids for arfolitixorin and leucovorin, the sequence of 5-FU and folate administration, the method of administration (bolus for arfolitixorin and infusion for leucovorin), and dosage of folate per cycle (120 mg/m^2^ for arfolitixorin vs. 400 mg/m^2^ for leucovorin). The leucovorin dose corresponds to 200 mg/m^2^ of the bioactive isomer ([6S]-5-formyl-tetrahydrofolate). However, we believe the potential for bias was mitigated with a stringent protocol and monitoring at study sites. In addition, the results are only generalizable to the sample enrolled, which comprised mainly patients of Caucasian ethnicity, and may not fully account for the known ethnic disparities in disease outcomes in colorectal cancer (28). The effect of the COVID-19 pandemic on death rates was not evaluated, although the small number of COVID-19–related major protocol deviations (5 patients, 1.0%) suggests it is unlikely to bias the treatment comparison. An advantage of this study was that there was no maximum age limit for patient eligibility, ensuring that the results are generalizable to older patients. This is important given that the incidence of mCRC increases with age (29).

In conclusion, this randomized phase III trial indicated that arfolitixorin (120 mg/m^2^) does not have superior efficacy to leucovorin (400 mg/m^2^) in mCRC, and it is possible that outcomes would be improved with a higher dose. The determinants of treatment response are also likely to be multifactorial, and there is a need for further investigation into biomarker-based predictors of response to promote a more patient-centered treatment approach.

## Supplementary Material

Supplementary Table 1 Supplementary Table 1. Detailed Inclusion and Exclusion criteria

Supplementary Table 2 Supplementary Table 2. Investigated Genes and Functions of the Corresponding Relevant Proteins

Supplementary Table 3 Supplementary Table 3. Comparison of Study Participants with Real-World Population

Supplementary Table 4 Supplementary Table 4. Patient Disposition

Supplementary Table 5 Supplementary Table 5. Interim Efficacy Results

Supplementary Table 6 Supplementary Table 6. Secondary Efficacy Endpoint: Progression-free Survival

Supplementary Table 7 Supplementary Table 7. Secondary Efficacy Endpoint: Duration of Response

Supplementary Table 8 Supplementary Table 8. Secondary Endpoint: Mean EQ VAS Scores Over Time

Supplementary Table 9 Supplementary Table 9. Grading of Adverse Events Reported in Any Category

Supplementary Table 10 Supplementary Table 10. Summary of Adverse Events

Supplementary Table 11 Supplementary Table 11. Serious Adverse Events

Supplementary Table 12 Supplementary Table 12. Adverse Events of Special Interest

Supplementary Table 13 Supplementary Table 13. Exploratory Endpoint: Recurrence-free Survival for Patients Undergoing Resective Surgery

Supplementary Table 14 Supplementary Table 14. Efficacy Outcomes in the Japanese Subgroup

Supplementary Table 15 Supplementary Table 15. Subgroup Analyses of Objective Response Rate (ORR)

Supplementary Figure 1 Kaplan–Meier Curve of Progression-Free Survival (key secondary endpoint) (ITT population)

Supplementary Figure 2 Kaplan–Meier Curve of Duration of Response (key secondary endpoint) (in patients with at least a partial response to treatment)

Supplementary Figure 3 Quality of Life Scores on EQ-5D for ‘usual activities’ over time (other secondary endpoint) (ITT population)

Supplementary Figure 4 Kaplan–Meier Curve of Recurrence-Free Survival for Patients Undergoing Resective Surgery (exploratory endpoint) (ITT population)

Supplementary Figure 5 Association between BRAF Expression and Progression-Free Survival (biomarker analysis set)

Supplementary Figure 6 Association between ERCC1 Expression and Progression-Free Survival (biomarker analysis set)

Supplementary Figure 7 Association between MTHFD2 Expression and Progression-Free Survival (biomarker analysis set)
