# Supplementary material for: A Randomized Phase III Study of Arfolitixorin versus Leucovorin with 5-Fluorouracil, Oxaliplatin, and Bevacizumab for First-Line Treatment of Metastatic Colorectal Cancer: The AGENT Trial
Source: Cancer Res Commun. 2024 Jan 4;4(1):28–37. doi: 10.1158/2767-9764.CRC-23-0361 (PMC10765772; doi:10.1158/2767-9764.CRC-23-0361)
Supplement: Supplementary Table 1 — Detailed Inclusion and Exclusion criteria [file crc-23-0361-s01.docx]

**Supplementary Table 1. Detailed Inclusion and Exclusion criteria**

| Participant inclusion criteria |
| --- |
| In order to be eligible to participate in the study, patients had to fulfill all of the following criteria:  1. Colorectal adenocarcinoma verified by biopsy;  2. Availability of biopsy material, from the primary tumor or metastasis, allowing for analysis of tumor gene expression;  3. Non-resectable, metastatic colorectal cancer (mCRC) planned for first-line therapy with 5-fluorouracil (5-FU), leucovorin (LV), oxaliplatin, and bevacizumab;  4. Evaluable disease with at least one measurable lesion of metastatic disease (≥10 mm in longest diameter on axial image on computed tomography (CT) scan or, alternatively, magnetic resonance imaging (MRI) with <5 mm reconstruction interval) or lymph node (≥15 mm in shortest axis when assessed by CT) within 28 days of randomization;  5. Life expectancy of more than 4 months;  6. ECOG (Eastern Cooperative Oncology Group) performance status 0 or 1;  7. Hemoglobin (Hb) >80 g/L, absolute neutrophil count (ANC) >1.5 x 10^9^/L, thrombocytes >100 x 10^9^/L;  8. Creatinine clearance >50 mL/min, total bilirubin <1.5 x the upper limit of normal (ULN), aspartate transaminase (AST) and alanine transaminase (ALT) <3 x ULN (and <5 x ULN in case of liver metastases);  9. Male or female ≥18 years of age;  10. Female patients of childbearing potential had to have a negative urine pregnancy test and use adequate contraceptive measures.^a^ Male patients had to use adequate contraceptive measures;^b^  11. Voluntarily signed informed consent form (ICF) before performance of any study-related procedure not part of normal medical care, with the understanding that consent may be withdrawn by the patient at any time without prejudice to future medical care.  ^a^Female patients had to be post-menopausal for more than 1 year or had to provide a negative pregnancy test and use an efficient method of contraception (i.e. a method with less than 1% failure rate [e.g. sterilization, hormone implants, hormone injections, some intrauterine devices, or vasectomized partner]) during the study and for 1 month (or more if requested by labels of products other than investigational medicinal product [IMP]) after the end of the study (last dose of IMP).  ^b^Unless the partner of a male patient was post-menopausal or using an efficient method of contraception as described above, male patients had to agree to use condoms during the study treatment and for 1 month after the end of the study treatment. |
| Participant exclusion criteria |
| Patients meeting any of the following criteria were ineligible to participate in the study:  1. Malignant tumors other than colorectal adenocarcinoma (current or within the previous 5 years), with the exception of curatively treated non-melanoma skin cancer or *in situ* carcinoma of the cervix;  2. Less than 6 months between randomization and completion of the last anticancer treatment (chemotherapy/radiotherapy/immunotherapy, etc.). NB: Rectal cancer treatment shorter than 8 weeks of chemo/radiation therapy was allowed;  3. Confirmation of progressive disease within 6 months after completion of prior adjuvant anticancer treatment;  4. Indication for any mCRC surgery or anticancer treatment other than study treatment;  5. Prior treatment with arfolitixorin;  6. Indication for treatment with a 5-FU analog, or 5-FU, for a condition other than mCRC;  7. Known dihydropyrimidine (DPD) deficiency;  8. Known or suspected central nervous system metastases;  9. Unresolved bowel obstruction, uncontrolled Crohn’s disease, or ulcerative colitis;  10. History of cardiac disease with a New York Heart Association class II or greater, congestive heart failure, myocardial infarction or unstable angina at any time during the 6 months prior to randomization, or serious arrhythmias requiring medication;  11. Current CTCAE (Common Terminology Criteria for Adverse Events) ≥ grade 3 diarrhea;  12. Current chronic infection or uncontrolled serious illness causing immunodeficiency;  13. Known or suspected hypersensitivity or intolerance to arfolitixorin, LV, 5-FU, oxaliplatin, or bevacizumab;  14. Breastfeeding patients;  15. Patients who received investigational drugs in other clinical trials within 28 days, or 5 half-lives of the investigational drug, prior to randomization;  16. Patients with serious medical or psychiatric illness likely to interfere with participation in this clinical study;  17. Ongoing drug or alcohol abuse, as deemed by the investigator;  18. Any condition that, in the opinion of the investigator, could compromise the patient's safety or adherence to the study protocol;  19. Involvement, or relation to people involved in the planning or conduct of the study (applied to both medical staff employed by the sponsor and staff at the study site);  20. Surgery (excluding previous diagnostic biopsy) in the 28-day period before randomization. |
