# Supplementary material for: A Randomized Phase III Study of Arfolitixorin versus Leucovorin with 5-Fluorouracil, Oxaliplatin, and Bevacizumab for First-Line Treatment of Metastatic Colorectal Cancer: The AGENT Trial
Source: Cancer Res Commun. 2024 Jan 4;4(1):28–37. doi: 10.1158/2767-9764.CRC-23-0361 (PMC10765772; doi:10.1158/2767-9764.CRC-23-0361)

**Supplementary Table 2. Investigated Genes and Functions of the Corresponding Relevant Proteins**


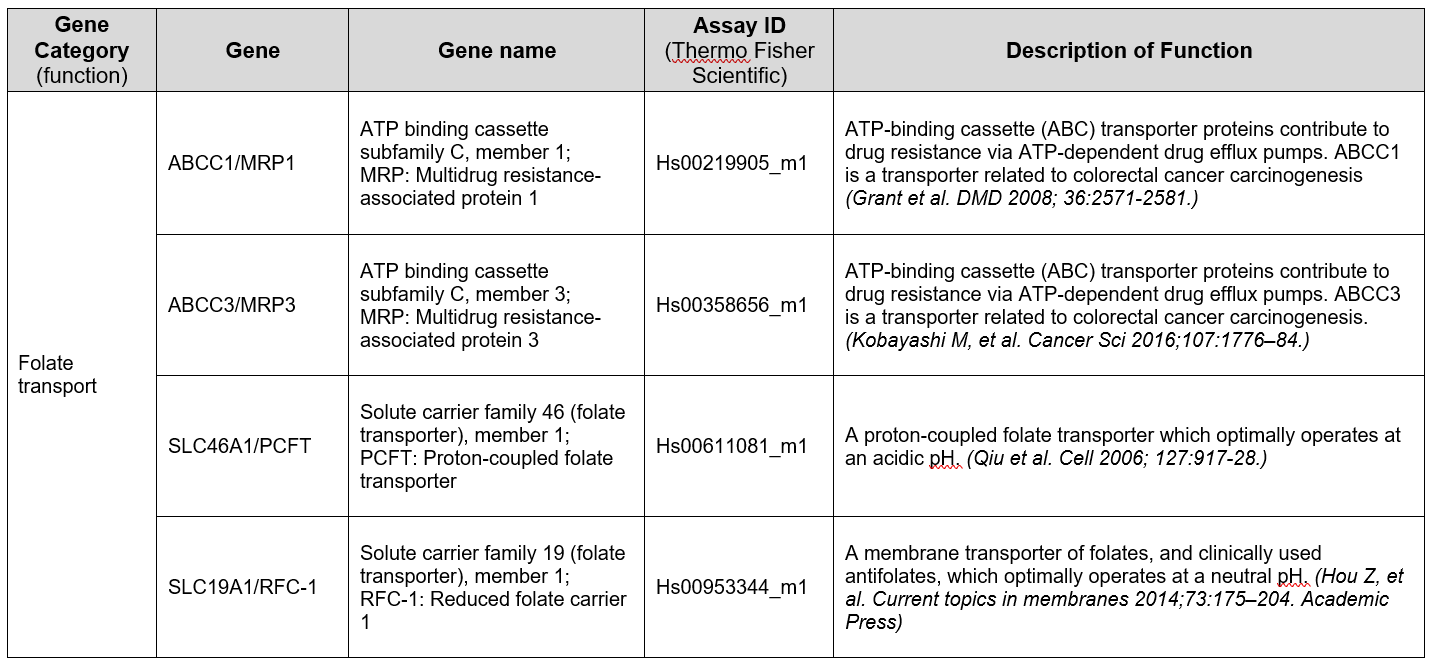


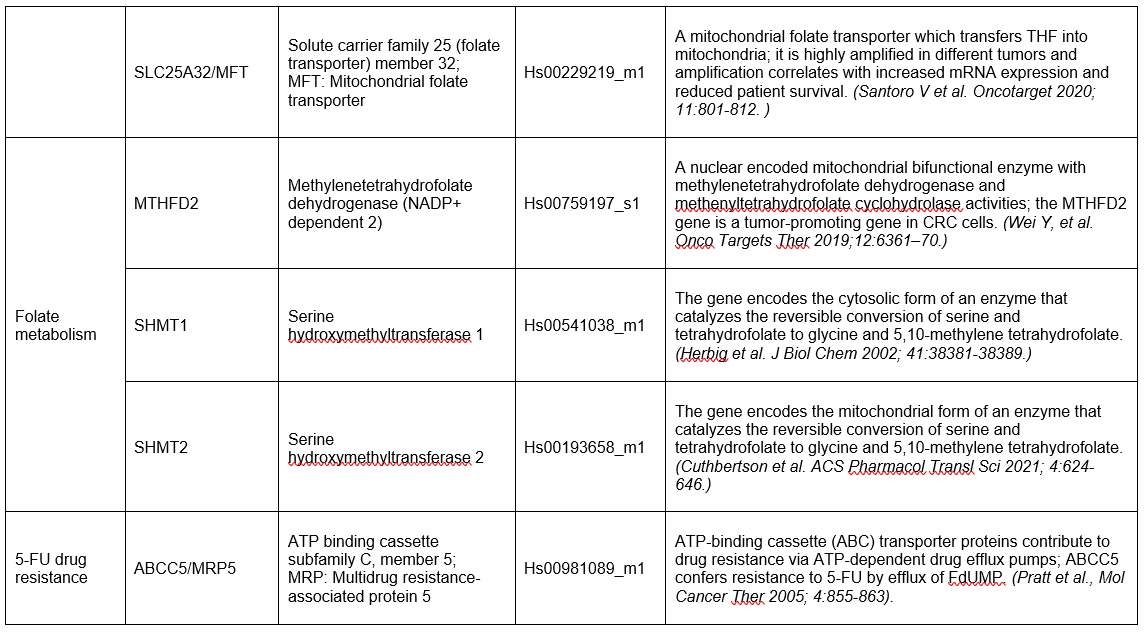


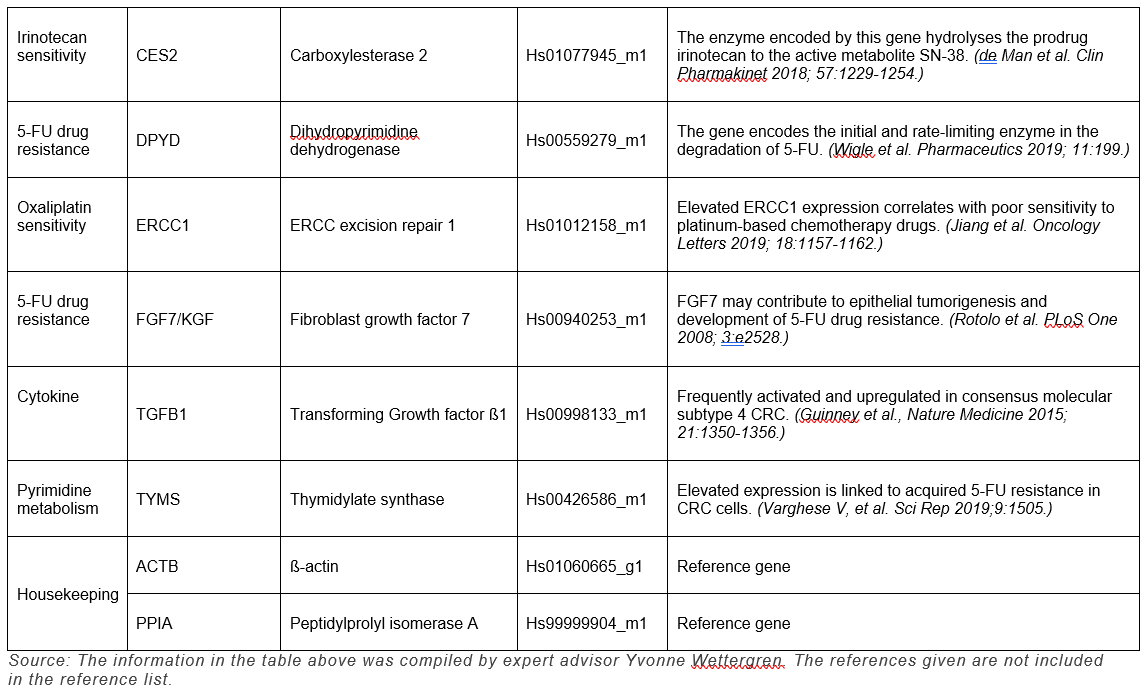

Supplement: Supplementary Table 2 — Investigated Genes and Functions of the Corresponding Relevant Proteins [file crc-23-0361-s02.docx]
