# Supplementary material for: A Randomized Phase III Study of Arfolitixorin versus Leucovorin with 5-Fluorouracil, Oxaliplatin, and Bevacizumab for First-Line Treatment of Metastatic Colorectal Cancer: The AGENT Trial
Source: Cancer Res Commun. 2024 Jan 4;4(1):28–37. doi: 10.1158/2767-9764.CRC-23-0361 (PMC10765772; doi:10.1158/2767-9764.CRC-23-0361)
Supplement: Supplementary Table 3 — Comparison of Study Participants with Real-World Population [file crc-23-0361-s03.docx]

**Supplementary Table 3. Comparison of Study Participants with Real-World Population**

| Cancer type(s)/subtype(s)/stage(s)/condition | Colorectal cancer (CRC); advanced metastatic disease |
| --- | --- |
| Considerations related to: |  |
| Sex | CRC is more common in males, with a 45% higher age-standardized incidence rate (~24 per 100,000 person-years) compared with females. |
| Age | CRC primarily impacts an older population, with most cases occurring in individuals aged ≥50 years. |
| Race/ethnicity | CRC affects people of all races. US data indicates that rates of early onset CRC are 21% higher in American Indians/Alaskan Natives, and 6% higher in black subjects, compared with White subjects. In the Hispanic population, CRC is the second most common type of cancer. |
| Geography | Globally, CRC is the third most prevalent cancer. In 2020, the global incidence of CRC was nearly 1.9 million. Incidence rates are highest in Europe, Australia, and New Zealand, and mortality rates highest in Eastern Europe. In high-income countries, incidence rates of CRC have declined, mainly due to effective screening. |
| Metastatic sites | The liver is the most common site of metastatic spread of CRC. |
| Genetic variants | Approximately 10% of CRC patients have mutant *BRAF*, 30–50% of patients have activating *KRAS* mutations, and ~7% of patients have mutations in *NRAS*. |
| Overall representativeness of this study | **Sex**  Over 60% of participants in this trial were male, which represents the higher incidence of CRC in males compared with females.  **Age**  The age range of the enrolled participants (55-71 years) was similar to the predominant age of occurrence of CRC (≥50 years).  **Race/ethnicity**  Most enrolled participants in this trial were White (73–78%). However, this trial also enrolled participants of Asian race (14–15%). There was limited representation of other races.  **Geography**  Patients were selected from 94 sites across 10 countries (Australia, Austria, Canada, France, Germany, Greece, Japan, Spain, Sweden, and the United States).  **Metastatic sites**  In this study, 72–75% of secondary metastases from a primary CRC tumor were reported in the liver.  **Genetic variants**  In this study, 9–17% of patients had mutations in *BRAF*, 62–66% of patients had activating *KRAS* mutations, and 10–16% of patients had mutant *NRAS*. |
