# Supplementary material for: A Randomized Phase III Study of Arfolitixorin versus Leucovorin with 5-Fluorouracil, Oxaliplatin, and Bevacizumab for First-Line Treatment of Metastatic Colorectal Cancer: The AGENT Trial
Source: Cancer Res Commun. 2024 Jan 4;4(1):28–37. doi: 10.1158/2767-9764.CRC-23-0361 (PMC10765772; doi:10.1158/2767-9764.CRC-23-0361)
Supplement: Supplementary Table 4 — Patient Disposition [file crc-23-0361-s04.docx]

**Supplementary Table 4. Patient Disposition**

| ***n* (%)** | **Arfolitixorin arm (n=245)** | **Leucovorin arm (n=245)** | **Total** |
| --- | --- | --- | --- |
| Number of patients screened |  |  | 594 |
| Number of patients not randomized |  |  | 104 |
| Patients randomized (ITT Population) | 245 (100%) | 245 (100) | 490 (82.5) |
| Patients in the Per-protocol Population | 141 (57.6) | 150 (61.2) | 291 (49.0) |
| Number of treated patients (Safety Population) | 243 | 238 | 481 |
| Treatment ongoing | 20 (8.2) | 24 (10.1) | 44 (9.1) |
| Treatment discontinued | 223 (91.8) | 214 (89.9) | 437 (90.9) |
| Primary reason for treatment discontinuation^a^ | | | |
| Withdrawal of consent | 14 (5.8) | 12 (5.0) | 26 (5.4) |
| Adverse event | 20 (8.2) | 19 (8.0) | 39 (8.1) |
| Protocol violation | 1 (0.4) | 1 (0.4) | 2 (0.4) |
| Non-compliance with study drug | 3 (1.2) | 3 (1.3) | 6 (1.2) |
| Physician decision | 32 (13.2) | 22 (9.2) | 54 (11.2) |
| Progressive disease | 126 (51.9) | 123 (51.7) | 249 (51.8) |
| Metastasis resection | 8 (3.3) | 13 (5.5) | 21 (4.4) |
| Other | 19 (7.8) | 21 (8.8) | 40 (8.3) |
| Number of patients who ended the study^b^ | 128 (52.2) | 117 (47.8) | 245 (50.0) |
| Screen failure | 0 | 5 (2.0) | 5 (1.0) |
| Withdrawal of consent | 6 (2.4) | 12 (4.9) | 18 (3.7) |
| Adverse event | 0 | 1 (0.4) | 1 (0.2) |
| Protocol violation | 1 (0.4) | 0 | 1 (0.2) |
| Death | 119 (48.6) | 99 (40.4) | 218 (44.5) |
| Physician decision | 2 (0.8) | 0 | 2 (0.4) |
| Pharmacokinetic analysis set^b^ | 19 (7.8) | 19 (7.8) | 38 (7.8) |

Abbreviation: ITT, intent-to-treat.

^a^Percentages based on the Safety Population.

^b^Percentages based on the ITT Population.
