# Supplementary material for: A Randomized Phase III Study of Arfolitixorin versus Leucovorin with 5-Fluorouracil, Oxaliplatin, and Bevacizumab for First-Line Treatment of Metastatic Colorectal Cancer: The AGENT Trial
Source: Cancer Res Commun. 2024 Jan 4;4(1):28–37. doi: 10.1158/2767-9764.CRC-23-0361 (PMC10765772; doi:10.1158/2767-9764.CRC-23-0361)
Supplement: Supplementary Table 5 — Interim Efficacy Results [file crc-23-0361-s05.docx]

**Supplementary Table 5. Interim Efficacy Results**

Data cut-off: January 12, 2021.

| ***N* (%)** | **Arfolitixorin arm** | **Leucovorin arm** | **Ratio** | ***P*-value** |
| --- | --- | --- | --- | --- |
| **ORR^a^** |  |  |  |  |
| *N* | 173 | 166 |  |  |
| *n* % [95% CI] | 83 (48.0%)  [40.3−55.6] | 72 (43.4%)  [35.7−51.2] | OR = 0.78  (0.20−3.00)^c^ | 0.7163^e^ |
|  |  |  |  |  |
| **Best overall response** |  |  |  |  |
| Complete response | 1 (0.6) | 1 (0.6) |  |  |
| Partial response | 82 (47.4) | 71 (42.8) |  |  |
| Stable disease | 75 (43.4) | 72 (43.4) |  |  |
| Progressive disease | 5 (2.9) | 7 (4.2) |  |  |
| Not evaluable | 1 (0.6) | 5 (3.0) |  |  |
| Non-CR/non-PD | 3 (1.7) | 2 (1.2) |  |  |
| Not done | 3 (1.7) | - |  |  |
| Subjects who discontinued | 3 (1.7) | 8 (4.8) |  |  |
|  |  |  |  |  |
| **PFS** |  |  |  |  |
| *N* | 228 | 227 |  |  |
| Number of events | 74 (32.5) | 71 (31.3) |  |  |
| Number of censored | 154 (67.5) | 156 (68.7) |  |  |
| Median (95% CI)^b^ | 11.1 (9.2−12.3) | 11.0 (9.0−12.0) | HR = 1.00 (0.71−1.41)^d^ | 0.98^f^ |
| 25% quartile (95% CI) | 7.4 (5.9−8.2) | 6.6 (5.7, 7.5) |  |  |
| 75% quartile (95% CI) | 14.8 (12.6−NR) | 15.9 (12.7−NR) |  |  |
| 6-months PFS rate, % (95% CI) | 81.1 (73.8−86.5) | 79.0 (71.3−84.8) |  |  |
| 12-months PFS rate, % (95% CI) | 41.5 (30.8−51.9) | 41.1 (30.3−51.5) |  |  |
| 18-months PFS rate, % (95% CI) | 11.5 (2.7−27.2) | 10.6 (0.9−33.9) |  |  |
| 24-months PFS rate, % (95% CI) | - | 0.0 |  |  |
|  |  |  |  |  |

Abbreviations: BICR, blinded independent central review; CI, confidence interval; CR, complete response; CRC, colorectal cancer; NR, not reached; ORR, overall response rate; PD, progressive disease; PFS, progression-free survival; PR, partial response; RECIST, Response Evaluation Criteria In Solid Tumors.

^a^Overall Response Rate is the proportion of patients who have a PR or CR according to RECIST v1.1. The ORR Interim Analysis Set corresponds to the subset of the Intent-to-treat Analysis Set for subjects who were evaluated by the BICR or who discontinued the study before any evaluation by the BICR.

^b^Based on Kaplan–Meier product limit estimates method.

^c^Mantel-Haenszel estimate of the common odds ratio and exact 95% two-sided confidence interval. The stratification factors are Geographic region, Tumor location and previous neo-adjuvant:adjuvant CRC treatment.

^d^Hazard ratio and 95% CI from a stratified Cox regression analysis with treatment as the sole explanatory variable.

^e^*P*-value from the stratified Cochran Mantel-Haenszel Chi-Squared test.

^f^*P*-value is based on the stratified log-rank test.
