# Supplementary material for: A Randomized Phase III Study of Arfolitixorin versus Leucovorin with 5-Fluorouracil, Oxaliplatin, and Bevacizumab for First-Line Treatment of Metastatic Colorectal Cancer: The AGENT Trial
Source: Cancer Res Commun. 2024 Jan 4;4(1):28–37. doi: 10.1158/2767-9764.CRC-23-0361 (PMC10765772; doi:10.1158/2767-9764.CRC-23-0361)
Supplement: Supplementary Table 6 — Secondary Efficacy Endpoint: Progression-free Survival [file crc-23-0361-s06.docx]

**Supplementary Table 6. Secondary Efficacy Endpoint: Progression-free Survival**

| **PFS Estimates** | **Arfolitixorin arm (*N* = 245)** | **Leucovorin arm (*N* = 245)** |
| --- | --- | --- |
| Median PFS, months (95% CI) | 12.8 (10.9–13.2) | 11.6 (11.0–14.5) |
| Number of events, *n* (%) | 132 (53.9) | 124 (50.6) |
| Progressive Disease | 120 (49.0) | 111 (45.3) |
| Death | 12 (4.9) | 13 (5.3) |
| New anti-cancer treatment | - | - |
| Number censored, *n* (%) | 113 (46.1) | 121 (49.4%) |
| 6-month PFS rate, % (95% CI) | 84.0 (78.4–88.2) | 81.6 (75.5–86.3) |
| 12-month PFS rate, % (95% CI) | 52.7 (45.0–59.8) | 49.0 (41.1–56.4) |
| 18-month PFS rate, % (95% CI) | 24.9 (17.5–32.9) | 30.6 (22.9–38.5) |
| 24-month PFS rate, % (95% CI) | 15.8 (9.3–23.8) | 17.5 (9.8–27.1) |

CI, confidence interval; ITT, intent-to-treat; PFS, progression-free survival.
