# Supplementary material for: A Randomized Phase III Study of Arfolitixorin versus Leucovorin with 5-Fluorouracil, Oxaliplatin, and Bevacizumab for First-Line Treatment of Metastatic Colorectal Cancer: The AGENT Trial
Source: Cancer Res Commun. 2024 Jan 4;4(1):28–37. doi: 10.1158/2767-9764.CRC-23-0361 (PMC10765772; doi:10.1158/2767-9764.CRC-23-0361)
Supplement: Supplementary Table 7 — Secondary Efficacy Endpoint: Duration of Response [file crc-23-0361-s07.docx]

**Supplementary Table 7. Secondary Efficacy Endpoint: Duration of Response**

| **DoR Estimates** | **Arfolitixorin arm (*N* = 118)** | **Leucovorin arm (*N* = 121)** |
| --- | --- | --- |
| Median DoR, months (95% CI) | 12.2 (11.1, 14.1) | 12.9 (10.6, 15.0) |
| Number of events, *n* (%) | 61 (51.7) | 63 (52.1) |
| Progressive Disease | 55 (46.6) | 61 (50.4) |
| Death | 6 (5.1) | 2 (1.7) |
| Number censored, *n* (%) | 57 (48.3) | 58 (47.9) |
| 6-month DoR rate, % (95% CI) | 82.1 (73.1–88.4) | 84.3 (76.0–90.0) |
| 12-month DoR rate, % (95% CI) | 54.3 (42.9–64.4) | 55.7 (45.0–65.2) |
| 18-month DoR rate, % (95% CI) | 27.4 (16.8–39.1) | 31.0 (19.8–42.8) |
| 24-month DoR rate, % (95% CI) | 16.2 (7.0–28.9) | 13.8 (4.3–28.6) |

Abbreviations: CI, confidence interval; DoR, duration of response.
