# Supplementary material for: A Randomized Phase III Study of Arfolitixorin versus Leucovorin with 5-Fluorouracil, Oxaliplatin, and Bevacizumab for First-Line Treatment of Metastatic Colorectal Cancer: The AGENT Trial
Source: Cancer Res Commun. 2024 Jan 4;4(1):28–37. doi: 10.1158/2767-9764.CRC-23-0361 (PMC10765772; doi:10.1158/2767-9764.CRC-23-0361)
Supplement: Supplementary Table 8 — Secondary Endpoint: Mean EQ VAS Scores Over Time [file crc-23-0361-s08.docx]

**Supplementary Table 8. Secondary Endpoint: Mean EQ VAS Scores Over Time**

| **Visit** | **Arfolitixorin arm (*N* = 245)** | **Leucovorin arm (*N* = 245)** |
| --- | --- | --- |
| Baseline | | |
| *N* | 233 | 228 |
| Mean (SD) | 74.5 (19.64) | 74.3 (19.19) |
| Week 8 | | |
| *N* | 196 | 193 |
| Mean (SD) | 75.9 (18.13) | 78.4 (16.66) |
| Week 16 | | |
| *N* | 184 | 178 |
| Mean (SD) | 74.9 (18.48) | 77.0 (17.38) |
| Week 24 | | |
| *N* | 167 | 147 |
| Mean (SD) | 74.8 (18.15) | 79.3 (15.20) |
| Week 32 | | |
| *N* | 122 | 121 |
| Mean (SD) | 75.0 (18.71) | 77.8 (17.10) |
| Week 40 | | |
| *N* | 92 | 93 |
| Mean (SD) | 76.4 (18.65) | 77.7 (17.34) |
| Week 48 | | |
| *N* | 73 | 74 |
| Mean (SD) | 77.8 (17.11) | 80.4 (17.45) |
| Week 56 | | |
| *N* | 56 | 56 |
| Mean (SD) | 80.0 (12.70) | 81.0 (18.85) |
| Week 64 | | |
| *N* | 36 | 48 |
| Mean (SD) | 78.9 (14.89) | 81.5 (16.14) |
| Week 72 | | |
| *N* | 24 | 26 |
| Mean (SD) | 81.1 (10.03) | 82.0 (14.47) |
| Week 80 | | |
| *N* | 18 | 18 |
| Mean (SD) | 83.4 (11.70) | 79.8 (17.29) |
| Week 88 | | |
| *N* | 14 | 12 |
| Mean (SD) | 85.6 (7.79) | 85.4 (15.26) |
| Week 96 | | |
| *N* | 13 | 9 |
| Mean (SD) | 86.5 (9.73) | 80.0 (18.65) |
| Week 104 | | |
| *N* | 10 | 5 |
| Mean (SD) | 86.0 (8.10) | 77.8 (22.57) |
| Week 112 | | |
| *N* | 4 | 3 |
| Mean (SD) | 80.0 (10.80) | 91.3 (14.15) |
| Week 120 | | |
| N | 3 | 3 |
| Mean (SD) | 83.7 (10.26) | 73.3 (22.55) |
| Week 128 | | |
| *N* | 1 | 2 |
| Mean (SD) | 70.0 | 70.0 (14.14) |
| Week 136 | | |
| *N* | 1 | 1 |
| Mean (SD) | 75.0 | 80.0 |
| End of treatment | | |
| *N* | 177 | 169 |
| Mean (SD) | 69.6 (20.42) | 73.1 (19.34) |

Abbreviation: SD, standard deviation.
