# Supplementary material for: A Randomized Phase III Study of Arfolitixorin versus Leucovorin with 5-Fluorouracil, Oxaliplatin, and Bevacizumab for First-Line Treatment of Metastatic Colorectal Cancer: The AGENT Trial
Source: Cancer Res Commun. 2024 Jan 4;4(1):28–37. doi: 10.1158/2767-9764.CRC-23-0361 (PMC10765772; doi:10.1158/2767-9764.CRC-23-0361)
Supplement: Supplementary Table 9 — Grading of Adverse Events Reported in Any Category [file crc-23-0361-s09.docx]

**Supplementary Table 9. Grading of Adverse Events Reported in Any Category**

| **All System Organ Classes** | **Arfolitixorin arm**  **(*N* = 243)** | **Leucovorin arm**  **(*N* = 238)** |
| --- | --- | --- |
| AEs overall |  |  |
| Patients with at least one AE, *n* (%) | 241 (99.2) | 236 (99.2) |
| Grade 1 | 5 (2.1) | 9 (3.8) |
| Grade 2 | 69 (28.4) | 67 (28.2) |
| Grade 3 | 126 (51.9) | 133 (55.9) |
| Grade 4 | 33 (13.6) | 19 (8.0) |
| Grade 5 | 8 (3.3) | 8 (3.4) |
| Total number of AEs | 2,998 | 2,872 |
| AEs considered to be related to the IMP |  |  |
| Patients with at least one AE, *n* (%) | 114 (46.9) | 103 (43.3) |
| Grade 1 | 45 (18.5) | 41 (17.2) |
| Grade 2 | 33 (13.6) | 36 (15.1) |
| Grade 3 | 29 (11.9) | 23 (9.7) |
| Grade 4 | 6 (2.5) | 3 (1.3) |
| Grade 5 | 1 (0.4) | 0 |
| Total number of AEs | 414 | 348 |

Abbreviations: AE, adverse event; IMP, investigational medicinal product**.**
