# Supplementary material for: A Randomized Phase III Study of Arfolitixorin versus Leucovorin with 5-Fluorouracil, Oxaliplatin, and Bevacizumab for First-Line Treatment of Metastatic Colorectal Cancer: The AGENT Trial
Source: Cancer Res Commun. 2024 Jan 4;4(1):28–37. doi: 10.1158/2767-9764.CRC-23-0361 (PMC10765772; doi:10.1158/2767-9764.CRC-23-0361)
Supplement: Supplementary Table 10 — Summary of Adverse Events [file crc-23-0361-s10.docx]

**Supplementary Table 10. Summary of Adverse Events**

| **Adverse event category, *n* (%)** | **Arfolitixorin arm (*N* = 243)** | **Leucovorin arm**  **(*N* = 238)** |
| --- | --- | --- |
| Any adverse event | 241 (99.2) | 236 (99.2) |
| With definite relationship to IMP | 114 (46.9) | 103 (43.3) |
| With definite relationship to the study procedure | 72 (29.6) | 61 (25.6) |
| With definite relationship to bevacizumab | 177 (72.8) | 169 (71.0) |
| With definite relationship to oxaliplatin | 237 (97.5) | 226 (95.0) |
| With definite relationship to 5-FU | 229 (94.2) | 215 (90.3) |
| Any serious adverse events | 81 (33.3) | 86 (36.1) |
| With definite relationship to IMP | 8 (3.3) | 8 (3.4) |
| Any grade 3, 4 or 5 adverse event | 167 (68.7) | 160 (67.2) |
| With definite relationship to IMP | 36 (14.8) | 26 (10.9) |
| Any adverse event leading to stop of study treatment | 5 (2.1) | 3 (1.3) |
| Any adverse event leading to death | 8 (3.3) | 8 (3.4) |
| Any adverse event related to IMP leading to death | 1 (0.4) | 0 |
| Any adverse event or death leading to study treatment discontinuation | 12 (4.9) | 11 (4.6) |
| Any adverse events of special interest | 180 (74.1) | 171 (71.8) |
| Any adverse event with frequency ≥10% | 239 (98.4) | 231 (97.1) |
| Any adverse event with frequency ≥10%  related to IMP | 96 (39.5) | 88 (37.0) |

Abbreviations: 5-FU, 5-fluorouracil; IMP, investigational medicinal product**.**
