# Supplementary material for: A Randomized Phase III Study of Arfolitixorin versus Leucovorin with 5-Fluorouracil, Oxaliplatin, and Bevacizumab for First-Line Treatment of Metastatic Colorectal Cancer: The AGENT Trial
Source: Cancer Res Commun. 2024 Jan 4;4(1):28–37. doi: 10.1158/2767-9764.CRC-23-0361 (PMC10765772; doi:10.1158/2767-9764.CRC-23-0361)
Supplement: Supplementary Table 11 — Serious Adverse Events [file crc-23-0361-s11.docx]

**Supplementary Table 11. Serious Adverse Events**

| **System Organ Class and Preferred Term** | **Arfolitixorin arm**  **(*N* = 243)** | **Leucovorin arm**  **(*N* = 238)** |
| --- | --- | --- |
| All System Organ Classes, *n* (%) | | |
| Patients with at least one AE | 81 (33.3) | 86 (36.1) |
| Total number of AEs | 145 | 120 |
| Gastrointestinal disorders, *n* (%) | | |
| Patients with at least one AE | 36 (14.8) | 35 (14.7) |
| Intestinal obstruction | 6 (2.5) | 10 (4.2) |
| Abdominal pain | 2 (0.8) | 7 (2.9) |
| Large intestinal obstruction | 5 (2.1) | 4 (1.7) |
| Small intestinal obstruction | 3 (1.2) | 5 (2.1) |
| Large intestine perforation | 3 (1.2) | 2 (0.8) |
| Colitis | 2 (0.8) | 1 (0.4) |
| Diarrhea | 2 (0.8) | 1 (0.4) |
| Rectal hemorrhage | 1 (0.4) | 2 (0.8) |
| Abdominal pain upper | 2 (0.8) | 0 |
| Ileus | 2 (0.8) | 0 |
| Abdominal hernia | 0 | 1 (0.4) |
| Crohn's disease | 1 (0.4) | 0 |
| Enterocolitis | 1 (0.4) | 0 |
| Gastrointestinal hemorrhage | 0 | 1 (0.4) |
| Inguinal hernia | 0 | 1 (0.4) |
| Intestinal perforation | 1 (0.4) | 0 |
| Intra-abdominal hemorrhage | 1 (0.4) | 0 |
| Lower gastrointestinal hemorrhage | 1 (0.4) | 0 |
| Melena | 1 (0.4) | 0 |
| Nausea | 0 | 1 (0.4) |
| Pancreatitis | 0 | 1 (0.4) |
| Pancreatitis acute | 1 (0.4) | 0 |
| Rectal perforation | 0 | 1 (0.4) |
| Subileus | 1 (0.4) | 0 |
| Vomiting | 1 (0.4) | 0 |
| Total number of AEs | 37 | 38 |
| Infections and infestations, *n* (%) | | |
| Patients with at least one AE | 22 (9.1) | 22 (9.2) |
| Device-related infection | 3 (1.2) | 3 (1.3) |
| Sepsis | 3 (1.2) | 3 (1.3) |
| Urinary tract infection | 1 (0.4) | 4 (1.7) |
| Corona virus infection | 3 (1.2) | 1 (0.4) |
| Pneumonia | 3 (1.2) | 1 (0.4) |
| Septic shock | 1 (0.4) | 1 (0.4) |
| Urosepsis | 0 | 2 (0.8) |
| Abdominal abscess | 1 (0.4) | 0 |
| Abdominal sepsis | 1 (0.4) | 0 |
| Abdominal wall abscess | 1 (0.4) | 0 |
| Appendicitis | 0 | 1 (0.4) |
| Bacteremia | 0 | 1 (0.4) |
| Biliary tract infection | 0 | 1 (0.4) |
| *Clostridium difficile* infection | 1 (0.4) | 0 |
| Ecthyma | 0 | 1 (0.4) |
| Enterococcal infection | 1 (0.4) | 0 |
| Epididymitis | 0 | 1 (0.4) |
| Escherichia infection | 1 (0.4) | 0 |
| Influenza | 0 | 1 (0.4) |
| Intestinal fistula infection | 1 (0.4) | 0 |
| *Klebsiella* infection | 1 (0.4) | 0 |
| Liver abscess | 0 | 1 (0.4) |
| Pelvic abscess | 0 | 1 (0.4) |
| Peritonitis | 1 (0.4) | 0 |
| Pneumonia viral | 1 (0.4) | 0 |
| Pulmonary sepsis | 1 (0.4) | 0 |
| Pyelonephritis | 1 (0.4) | 0 |
| Respiratory tract infection | 1 (0.4) | 0 |
| Tracheobronchitis | 0 | 1 (0.4) |
| Total number of AEs | 27 | 24 |
| Respiratory, thoracic, and mediastinal disorders, *n* (%) | | |
| Patients with at least one AE | 16 (6.6) | 12 (5.0) |
| Pulmonary embolism | 8 (3.3) | 8 (3.4) |
| Respiratory failure | 2 (0.8) | 1 (0.4) |
| Dyspnea | 1 (0.4) | 1 (0.4) |
| Bronchial hyperreactivity | 0 | 1 (0.4) |
| Dyspnea exertional | 1 (0.4) | 0 |
| Hemoptysis | 1 (0.4) | 0 |
| Interstitial lung disease | 1 (0.4) | 0 |
| Lung disorder | 1 (0.4) | 0 |
| Pleural effusion | 1 (0.4) | 0 |
| Pneumothorax | 0 | 1 (0.4) |
| Total number of AEs | 16 | 12 |
| General disorders and administration site conditions, *n* (%) | | |
| Patients with at least one AE | 9 (3.7) | 7 (2.9) |
| Pyrexia | 6 (2.5) | 3 (1.3) |
| General physical health deterioration | 1 (0.4) | 3 (1.3) |
| Catheter site extravasation | 1 (0.4) | 0 |
| Chest pain | 0 | 1 (0.4) |
| Condition aggravated | 1 (0.4) | 0 |
| Mucosal inflammation | 0 | 1 (0.4) |
| Total number of AEs | 9 | 8 |
| Blood and lymphatic system disorders, *n* (%) | | |
| Patients with at least one AE | 6 (2.5) | 7 (2.9) |
| Febrile neutropenia | 3 (1.2) | 5 (2.1) |
| Anemia | 2 (0.8) | 1 (0.4) |
| Hemolytic anemia | 1 (0.4) | 0 |
| Leukemoid reaction | 0 | 1 (0.4) |
| Pancytopenia | 1 (0.4) | 0 |
| Total number of AEs | 7 | 7 |
| Injury, poisoning, and procedural complications, *n* (%) | | |
| Patients with at least one AE | 8 (3.3) | 5 (2.1) |
| Fall | 1 (0.4) | 1 (0.4) |
| Hip fracture | 1 (0.4) | 1 (0.4) |
| Gastrointestinal anastomotic leak | 0 | 1 (0.4) |
| Gastrointestinal stoma complication | 1 (0.4) | 0 |
| Infusion-related reaction | 1 (0.4) | 0 |
| Pelvic fracture | 0 | 1 (0.4) |
| Post-procedural complication | 1 (0.4) | 0 |
| Rib fracture | 1 (0.4) | 0 |
| Road traffic accident | 1 (0.4) | 0 |
| Skin laceration | 1 (0.4) | 0 |
| Stoma site hemorrhage | 0 | 1 (0.4) |
| Wound dehiscence | 1 (0.4) | 0 |
| Total number of AEs | 9 | 5 |
| Renal and urinary disorders, *n* (%) | | |
| Patients with at least one AE | 5 (2.1) | 5 (2.1) |
| Acute kidney injury | 2 (0.8) | 3 (1.3) |
| Urinary retention | 1 (0.4) | 2 (0.8) |
| Renal colic | 1 (0.4) | 0 |
| Ureterolithiasis | 1 (0.4) | 0 |
| Total number of AEs | 5 | 5 |
| Cardiac disorders, *n* (%) | | |
| Patients with at least one AE | 6 (2.5) | 3 (1.3) |
| Atrial fibrillation | 1 (0.4) | 1 (0.4) |
| Acute left ventricular failure | 0 | 1 (0.4) |
| Acute myocardial infarction | 1 (0.4) | 0 |
| Atrial thrombosis | 0 | 1 (0.4) |
| Bradycardia | 1 (0.4) | 0 |
| Cardiac failure acute | 1 (0.4) | 0 |
| Cardio-respiratory arrest | 1 (0.4) | 0 |
| Coronary artery disease | 1 (0.4) | 0 |
| Low cardiac output syndrome | 1 (0.4) | 0 |
| Myocardial infarction | 1 (0.4) | 0 |
| Total number of AEs | 8 | 3 |
| Metabolism and nutrition disorders, *n* (%) | | |
| Patients with at least one AE | 7 (2.9) | 2 (0.8) |
| Hyponatremia | 2 (0.8) | 1 (0.4) |
| Acidosis hyperchloremic | 1 (0.4) | 0 |
| Diabetes mellitus | 1 (0.4) | 0 |
| Diabetic ketoacidosis | 0 | 1 (0.4) |
| Diabetic metabolic decompensation | 1 (0.4) | 0 |
| Hypokalemia | 1 (0.4) | 0 |
| Lactic acidosis | 1 (0.4) | 0 |
| Total number of AEs | 7 | 2 |
| Nervous system disorders, *n* (%) | | |
| Patients with at least one AE | 7 (2.9) | 2 (0.8) |
| Syncope | 2 (0.8) | 1 (0.4) |
| Encephalopathy | 1 (0.4) | 0 |
| Hypersomnia | 1 (0.4) | 0 |
| Lethargy | 1 (0.4) | 0 |
| Metabolic encephalopathy | 1 (0.4) | 0 |
| Myoclonus | 1 (0.4) | 0 |
| Peroneal nerve palsy | 1 (0.4) | 0 |
| Status epilepticus | 1 (0.4) | 0 |
| Transient ischemic attack | 0 | 1 (0.4) |
| Total number of AEs | 9 | 2 |
| Hepatobiliary disorders, *n* (%) | | |
| Patients with at least one AE | 2 (0.8) | 5 (2.1) |
| Cholangitis | 0 | 2 (0.8) |
| Biliary fistula | 1 (0.4) | 0 |
| Cholelithiasis | 0 | 1 (0.4) |
| Hepatic function abnormal | 0 | 1 (0.4) |
| Jaundice | 1 (0.4) | 0 |
| Portosplenomesenteric venous thrombosis | 0 | 1 (0.4) |
| Total number of AEs | 2 | 5 |
| Vascular disorders, *n* (%) | | |
| Patients with at least one AE | 4 (1.6) | 2 (0.8) |
| Deep vein thrombosis | 2 (0.8) | 0 |
| Embolism arterial | 0 | 1 (0.4) |
| Hypertension | 0 | 1 (0.4) |
| Hypotension | 1 (0.4) | 0 |
| Superior vena cava syndrome | 1 (0.4) | 0 |
| Total number of AEs | 4 | 2 |
| Neoplasms benign, malignant, and unspecified (including cysts and polyps), *n* (%) | | |
| Patients with at least one AE | 1 (0.4) | 2 (0.8) |
| Lung neoplasm malignant | 0 | 1 (0.4) |
| Prostate cancer | 1 (0.4) | 0 |
| Tumor perforation | 0 | 1 (0.4) |
| Total number of AEs | 1 | 2 |
| Investigations, *n* (%) | | |
| Patients with at least one AE | 2 (0.8) | 0 |
| C-reactive protein increased | 1 (0.4) | 0 |
| Neutrophil count decreased | 1 (0.4) | 0 |
| Total number of AEs | 2 | 0 |
| Musculoskeletal and connective tissue disorders, *n* (%) | | |
| Patients with at least one AE | 1 (0.4) | 1 (0.4) |
| Back pain | 0 | 1 (0.4) |
| Flank pain | 1 (0.4) | 0 |
| Total number of AEs | 1 | 1 |
| Congenital, familial, and genetic disorders, *n* (%) | | |
| Patients with at least one AE | 0 | 1 (0.4) |
| Hypertrophic cardiomyopathy | 0 | 1 (0.4) |
| Total number of AEs | 0 | 1 |
| Eye disorders, *n* (%) | | |
| Patients with at least one AE | 0 | 1 (0.4) |
| Retinal detachment | 0 | 1 (0.4) |
| Total number of AEs | 0 | 1 |
| Immune system disorders, *n* (%) | | |
| Patients with at least one AE | 0 | 1 (0.4) |
| Anaphylactic shock | 0 | 1 (0.4) |
| Total number of AEs | 0 | 1 |
| Reproductive system and breast disorders, *n* (%) | | |
| Patients with at least one AE | 1 (0.4) | 0 |
| Rectoprostatic fistula | 1 (0.4) | 0 |
| Total number of AEs | 1 | 0 |
| Skin and subcutaneous tissue disorders, *n* (%) | | |
| Patients with at least one AE | 0 | 1 (0.4) |
| Night sweats | 0 | 1 (0.4) |
| Total number of AEs | 0 | 1 |

Abbreviation: AE, adverse event.
