# Supplementary material for: A Randomized Phase III Study of Arfolitixorin versus Leucovorin with 5-Fluorouracil, Oxaliplatin, and Bevacizumab for First-Line Treatment of Metastatic Colorectal Cancer: The AGENT Trial
Source: Cancer Res Commun. 2024 Jan 4;4(1):28–37. doi: 10.1158/2767-9764.CRC-23-0361 (PMC10765772; doi:10.1158/2767-9764.CRC-23-0361)
Supplement: Supplementary Table 12 — Adverse Events of Special Interest [file crc-23-0361-s12.docx]

**Supplementary Table 12. Adverse Events of Special Interest**

| **System Organ Class and Preferred Term** | **Arfolitixorin**  **(*N* = 243)** | **Leucovorin**  **(*N* = 238)** |
| --- | --- | --- |
| All System Organ Classes | | |
| Patients with at least one AE,  *n* (%) | 180 (74.1) | 171 (71.8) |
| Grade 1 | 71 (29.2) | 68 (28.6) |
| Grade 2 | 62 (25.5) | 61 (25.6) |
| Grade 3 | 37 (15.2) | 35 (14.7) |
| Grade 4 | 10 (4.1) | 7 (2.9) |
| Total number of AEs | 283 | 275 |
| Gastrointestinal disorders, *n* (%) | | |
| Patients with at least one AE | 140 (57.6) | 139 (58.4) |
| Grade 1 | 88 (36.2) | 75 (31.5) |
| Grade 2 | 41 (16.9) | 53 (22.3) |
| Grade 3 | 11 (4.5) | 11 (4.6) |
| Total number of AEs | 170 | 175 |
| Diarrhea | 118 (48.6) | 113 (47.5) |
| Grade 1 | 83 (34.2) | 67 (28.2) |
| Grade 2 | 27 (11.1) | 36 (15.1) |
| Grade 3 | 8 (3.3) | 10 (4.2) |
| Stomatitis | 52 (21.4) | 62 (26.1) |
| Grade 1 | 34 (14.0) | 41 (17.2) |
| Grade 2 | 15 (6.2) | 20 (8.4) |
| Grade 3 | 3 (1.2) | 1 (0.4) |
| Blood and lymphatic system disorders, *n* (%) | | |
| Patients with at least one AE | 60 (24.7) | 51 (21.4) |
| Grade 1 | 8 (3.3) | 11 (4.6) |
| Grade 2 | 18 (7.4) | 15 (6.3) |
| Grade 3 | 24 (9.9) | 18 (7.6) |
| Grade 4 | 10 (4.1) | 7 (2.9) |
| Total number of AEs | 60 | 51 |
| Neutropenia | 60 (24.7) | 51 (21.4) |
| Grade 1 | 8 (3.3) | 11 (4.6) |
| Grade 2 | 18 (7.4) | 15 (6.3) |
| Grade 3 | 24 (9.9) | 18 (7.6) |
| Grade 4 | 10 (4.1) | 7 (2.9) |
| General disorders and administration site conditions, *n* (%) | | |
| Patients with at least one AE | 53 (21.8) | 49 (20.6) |
| Grade 1 | 34 (14.0) | 28 (11.8) |
| Grade 2 | 16 (6.6) | 14 (5.9) |
| Grade 3 | 3 (1.2) | 7 (2.9) |
| Total number of AEs | 53 | 49 |
| Mucosal inflammation | 53 (21.8) | 49 (20.6) |
| Grade 1 | 34 (14.0) | 28 (11.8) |
| Grade 2 | 16 (6.6) | 14 (5.9) |
| Grade 3 | 3 (1.2) | 7 (2.9) |

Abbreviation: AE, adverse event.
