# Supplementary material for: A Randomized Phase III Study of Arfolitixorin versus Leucovorin with 5-Fluorouracil, Oxaliplatin, and Bevacizumab for First-Line Treatment of Metastatic Colorectal Cancer: The AGENT Trial
Source: Cancer Res Commun. 2024 Jan 4;4(1):28–37. doi: 10.1158/2767-9764.CRC-23-0361 (PMC10765772; doi:10.1158/2767-9764.CRC-23-0361)
Supplement: Supplementary Table 13 — Exploratory Endpoint: Recurrence-free Survival for Patients Undergoing Resective Surgery [file crc-23-0361-s13.docx]

**Supplementary Table 13. Exploratory Endpoint: Recurrence-free Survival for Patients Undergoing Resective Surgery**

| **RFS Estimates** | **Arfolitixorin arm**  **(*N* = 18)** | **Leucovorin arm**  **(*N* = 15)** |
| --- | --- | --- |
| Median RFS, months (95% CI) | 10.6 (2.0–16.1) | 5.6 (2.1–NR) |
| Number of events, *n* (%) | 9 (50.0) | 8 (53.3) |
| Progressive Disease | 8 (44.4) | 8 (53.3) |
| Death | 1 (5.6) | - |
| Number censored, *n* (%) | 9 (50.0) | 7 (46.7) |
| 6-month RFS rate, % (95% CI) | 58.8 (29.3–79.5) | 41.1 (14.0–66.8) |
| 12-month RFS rate, % (95% CI) | 29.4 (5.2–60.2) | 30.8 (7.9–57.9) |
| 18-month RFS rate, % (95% CI) | 14.7 (0.8–46.4) | - |
| 24-month RFS rate, % (95% CI) | - | - |

Abbreviations: CI, confidence survival; NR, not reached; RFS, recurrence-free survival.
