# Supplementary material for: A Randomized Phase III Study of Arfolitixorin versus Leucovorin with 5-Fluorouracil, Oxaliplatin, and Bevacizumab for First-Line Treatment of Metastatic Colorectal Cancer: The AGENT Trial
Source: Cancer Res Commun. 2024 Jan 4;4(1):28–37. doi: 10.1158/2767-9764.CRC-23-0361 (PMC10765772; doi:10.1158/2767-9764.CRC-23-0361)
Supplement: Supplementary Table 14 — Efficacy Outcomes in the Japanese Subgroup [file crc-23-0361-s14.docx]

**Supplementary Table 14. Efficacy Outcomes in the Japanese Subgroup**

(a)

|  | **Arfolitixorin arm**  **(*n* = 29)** | **Leucovorin arm**  **(*n* = 29)** | **Risk difference**  **(95% CI)^a^** | ***P*-value** |
| --- | --- | --- | --- | --- |
| **ORR, *n* (%) [95% CI]** | 15 (51.7) [32.5−70.6] | 21 (72.4) [52.8−87.3] | −0.21  [−0.45 to −0.04] |  |
| Complete response, *n* (%) | - | 2 (6.9) |  |  |
| Partial response, *n* (%) | 15 (51.7) | 19 (65.5) |  |  |
| Stable disease, *n* (%) | 14 (48.3) | 8 (27.6) |  |  |

Abbreviations: CI, confidence interval; ORR, overall response rate.

^a^Mantel-Haenszel estimate of the common risk difference and 95% two-sided confidence interval.

(b)

| **ORR (%)** | **All randomized patients**  **(*n* = 490)** | **Japanese patients**  **(*n* = 58)** |
| --- | --- | --- |
| Arfolitixorin arm | 48.2 | 51.7 |
| Leucovorin arm | 49.4 | 72.4 |
| ORR risk difference^a^ | −0.009 | −0.207 |

Abbreviations: CRC, colorectal cancer; ORR, overall response rate.

^a^ORR risk difference for all randomized patients is estimated with stratification factors: Geographic Region, Primary Tumor Location, and Previous Adjuvant CRC Treatment. No stratification factor was considered for estimation of ORR risk difference for Japanese patients.

(c)

| **PFS, months (95% CI)** | **Arfolitixorin arm**  **(*n* = 29)** | **Leucovorin arm**  **(*n* = 29)** | **Hazard ratio** | ***P*-value** |
| --- | --- | --- | --- | --- |
| **Median^a^** | 12.9 (10.4−NR) | 14.7 (11.1−NR) | 1.386 (0.648−2.967)^b^ | 0.66 |
| 6-months PFS^a^ | 88.9 (69.4−96.3) | 84.7 (64.1−94.0) |  |  |
| 12-months PFS^a^ | 55.7 (34.3−72.7) | 62.1 (39.4−78.3) |  |  |
| 18-months PFS^a^ | 35.5 (16.6−55.0) | 44.3 (22.1−64.4) |  |  |
| 24-months PFS^a^ | 35.5 (16.6−55.0) | - |  |  |

Abbreviations: CI, confidence interval; NR, not reached; PFS, progression-free survival.

^a^Based on Kaplan–Meier product limit estimates method.

^b^Hazard ratio and 95% CI from a stratified Cox regression analysis with treatment as the main explanatory variable.

(d)

| **DoR, months (95% CI)** | **Arfolitixorin arm**  **(*n* = 15)** | **Leucovorin arm**  **(*n* = 21)** | **Hazard ratio** | ***P*-value** |
| --- | --- | --- | --- | --- |
| **Median^a^** | 11.1 (5.6−NR) | 12.9 (7.2−NR) | 1.334 (0.447−3.982)^b^ | 0.74 |
| 6-months DoR^a^ | 73.3 (43.6−89.1) | 83.0 (55.9−94.2) |  |  |
| 12-months DoR^a^ | 45.3 (16.6−70.4) | 68.5 (38.9−85.9) |  |  |
| 18-months DoR^a^ | 45.3 (16.6−70.4) | - |  |  |
| 24-months DoR^a^ | 45.3 (16.6−70.4) | - |  |  |

Abbreviations: CI, confidence interval; DoR, duration of response; NR, not reached.

^a^Based on Kaplan–Meier product limit estimates method.

^b^Hazard ratio and 95% CI from a stratified Cox regression analysis with treatment as the main explanatory variable.

(e)

| **RFS, months (95% CI)** | **Arfolitixorin arm**  **(*n* = 2)** | **Leucovorin arm**  **(*n* = 0)** | **Hazard ratio** | ***P*-value** |
| --- | --- | --- | --- | --- |
| **Median** | Not reached | - |  |  |
| 6-months RFS | - | - |  |  |
| 12-months RFS | - | - |  |  |
| 18-months RFS | - | - |  |  |
| 24-months RFS | - | - |  |  |

Abbreviations: CI, confidence interval; RFS, recurrence-free survival.

(f)

| **OS, % (95% CI)** | **Arfolitixorin**  **(*n* = 29)** | **Leucovorin**  **(*n* = 29)** | **Hazard ratio** | ***P*-value** |
| --- | --- | --- | --- | --- |
| **Median^a^** | NR (21.0−NR) | NR | 1.398 (0.386−5.069)^b^ | 0.67 |
| 6-months OS^a^ | 100.0 (100.0−100.0) | 100.0 (100.0−100.0) |  |  |
| 12-months OS^a^ | 85.7 (66.3−94.4) | 85.8 (66.4−94.4) |  |  |
| 18-months OS^a^ | 80.4 (58.3−91.5) | 85.8 (66.4−94.4) |  |  |
| 24-months OS^a^ | 64.3 (27.0−86.2) | 85.8 (66.4−94.4) |  |  |

Abbreviations: CI, confidence interval; NR, not reached; OS, overall survival.

^a^Based on Kaplan–Meier product limit estimates method.

^b^Hazard ratio and 95% CI from a stratified Cox regression analysis with treatment as the main explanatory variable.
