# Supplementary material for: A Randomized Phase III Study of Arfolitixorin versus Leucovorin with 5-Fluorouracil, Oxaliplatin, and Bevacizumab for First-Line Treatment of Metastatic Colorectal Cancer: The AGENT Trial
Source: Cancer Res Commun. 2024 Jan 4;4(1):28–37. doi: 10.1158/2767-9764.CRC-23-0361 (PMC10765772; doi:10.1158/2767-9764.CRC-23-0361)
Supplement: Supplementary Table 15 — Subgroup Analyses of Objective Response Rate (ORR) [file crc-23-0361-s15.docx]

**Supplementary Table 15. Subgroup Analyses of Objective Response Rate (ORR)**

| **Characteristic** | **Subgroup** | **Arfolitixorin arm** | | **Leucovorin arm** | |
| --- | --- | --- | --- | --- | --- |
|  |  | **N** | **ORR [95% CI]** | **N** | **ORR [95% CI]** |
| Primary tumor location | Right colon | 86 | 52.3% [41.2–63.2] | 82 | 46.3% [35.2–57.7] |
|  | Left colon | 84 | 52.4% [41.1–63.4] | 84 | 48.8% [37.7–59.9] |
|  | Rectum | 75 | 38.7% [27.6–50.6] | 79 | 53.2% [41.6–64.4] |
| Previous adjuvant therapy | No | 206 | 49.5% [42.4–56.5] | 208 | 49.5% [42.5–56.5] |
|  | Yes | 39 | 41.0% [25.5–57.9] | 37 | 48.6% [31.9–65.6] |
| Primary tumor resected | Yes | 110 | 52.7% [42.9–62.3] | 109 | 52.3% [42.5–61.9] |
|  | No | 135 | 44.4% [35.9–53.2] | 136 | 47.1% [38.4–55.8] |
| ECOG performance status | 0 | 163 | 47.9% [39.9–55.8] | 156 | 50.0% [41.9–58.1] |
|  | 1 | 79 | 49.4% [37.9–60.8] | 82 | 52.4% [41.1–63.5] |
|  | 2 | 1 | 100.0% [2.5–100.0] | 0 | - |
| *BRAF* status | Mutant | 18 | 22.2% [6.4–47.6] | 13 | 30.8% [9.0–61.4] |
|  | WT | 91 | 44.0% [33.5–54.7] | 118 | 50.8% [41.4–60.1] |
| *KRAS* status | Mutant | 101 | 45.5% [35.6–55.7] | 112 | 49.1% [39.5–58.7] |
|  | WT | 61 | 44.3% [31.5–57.5] | 59 | 45.8% [32.7–59.2] |
| *NRAS* status | Mutant | 16 | 50.0% [24.6–75.3] | 14 | 35.7% [12.7–64.8] |
|  | WT | 85 | 36.5% [26.2–47.6] | 114 | 49.1% [39.6–58.6] |
| Liver metastasis | Yes | 177 | 50.8% [43.2–58.4] | 184 | 49.5% [42.0–56.9] |
|  | No | 0 | - | 0 | - |
| Lung metastasis | Yes | 110 | 49.1% [39.4–58.8] | 119 | 52.9% [43.5–62.1] |
|  | No | 0 | - | 0 | - |
| Peritoneal metastasis | Yes | 41 | 39.0% [24.2–55.5] | 34 | 41.2% [24.6–59.3] |
|  | No | 0 | - | 0 | - |
| Other metastasis | Yes | 118 | 43.2% [34.1–52.6] | 194 | 48.9% [38.4–59.4] |
|  | No | 0 | - | 0 | - |
| Geographic region | Australia | 15 | 53.3% [26.5–78.7] | 16 | 56.3% [29.8–80.2] |
|  | Europe | 138 | 41.3% [33.0–49.9] | 135 | 43.0% [34.4–51.7] |
|  | Japan | 29 | 51.7% [32.5–70.5] | 29 | 72.4% [52.7–87.2] |
|  | North America | 63 | 60.3% [47.2–72.4] | 65 | 50.8% [38.0–63.4] |
| Sex | Male | 162 | 47.5% [39.6–55.5] | 151 | 47.0% [38.8–55.3] |
|  | Female | 83 | 49.4% [38.2–60.6] | 94 | 53.2% [42.6–63.5] |
| Age | <65 years | 139 | 47.5% [38.9–56.1] | 128 | 53.1% [44.1–62.0] |
|  | ≥65 years | 106 | 49.1% [39.2–58.9] | 117 | 45.3% [36.0–54.7] |
| Age | ≤75 years | 218 | 49.5% [42.7–56.3] | 213 | 50.2% [43.3–57.1] |
|  | >75 years | 27 | 37.0% [19.4–57.6] | 32 | 43.8% [26.3–62.3] |
| BMI | <25 kg/m^2^ | 115 | 50.4% [40.9–59.8] | 101 | 51.5% [41.3–61.5] |
|  | ≥25 kg/m^2^ | 128 | 46.9% [38.0–55.8] | 135 | 51.1% [42.3–59.8] |

Abbreviations: BMI, body mass index; CI, confidence interval; ECOG, Eastern Cooperative Oncology Group; ORR, overall response rate; WT, wild-type.
