# Supplementary material for: A Randomized Phase III Study of Arfolitixorin versus Leucovorin with 5-Fluorouracil, Oxaliplatin, and Bevacizumab for First-Line Treatment of Metastatic Colorectal Cancer: The AGENT Trial
Source: Cancer Res Commun. 2024 Jan 4;4(1):28–37. doi: 10.1158/2767-9764.CRC-23-0361 (PMC10765772; doi:10.1158/2767-9764.CRC-23-0361)
Supplement: Supplementary Figure 1 — Kaplan–Meier Curve of Progression-Free Survival (key secondary endpoint) (ITT population) [file crc-23-0361-s16.pdf]

Supplementary Figure 1. Kaplan–Meier Curve of Progression-Free Survival (key secondary endpoint) (ITT population)

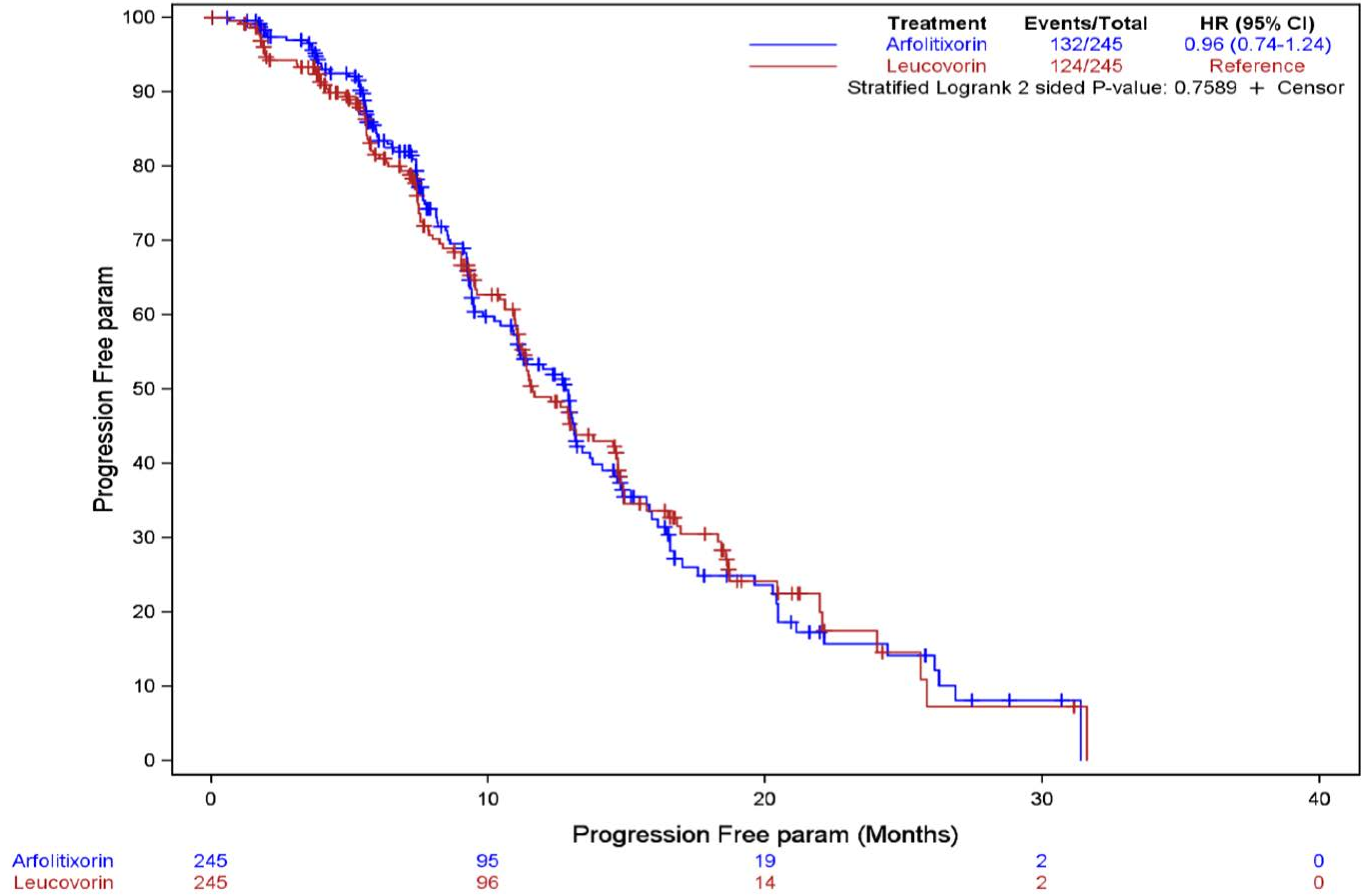

CI, confidence interval; HR, hazard ratio; ITT, intention-to-treat.
