# Supplementary material for: A Randomized Phase III Study of Arfolitixorin versus Leucovorin with 5-Fluorouracil, Oxaliplatin, and Bevacizumab for First-Line Treatment of Metastatic Colorectal Cancer: The AGENT Trial
Source: Cancer Res Commun. 2024 Jan 4;4(1):28–37. doi: 10.1158/2767-9764.CRC-23-0361 (PMC10765772; doi:10.1158/2767-9764.CRC-23-0361)
Supplement: Supplementary Figure 2 — Kaplan–Meier Curve of Duration of Response (key secondary endpoint) (in patients with at least a partial response to treatment) [file crc-23-0361-s17.pdf]

Supplementary Figure 2. Kaplan–Meier Curve of Duration of Response (key secondary endpoint) (in patients with at least a partial response to treatment)

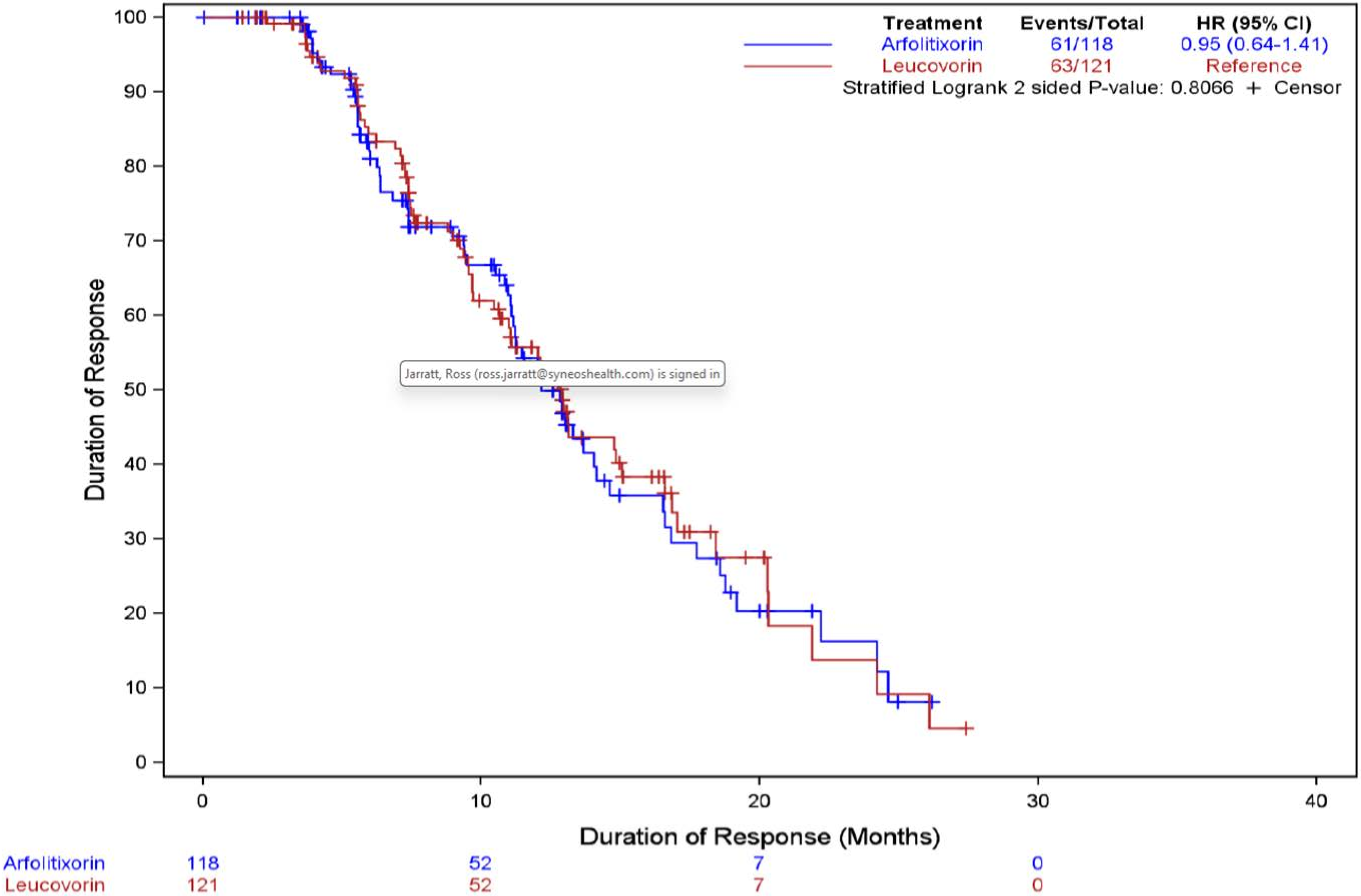

CI, confidence interval; HR, hazard ratio.
