# Supplementary material for: A Randomized Phase III Study of Arfolitixorin versus Leucovorin with 5-Fluorouracil, Oxaliplatin, and Bevacizumab for First-Line Treatment of Metastatic Colorectal Cancer: The AGENT Trial
Source: Cancer Res Commun. 2024 Jan 4;4(1):28–37. doi: 10.1158/2767-9764.CRC-23-0361 (PMC10765772; doi:10.1158/2767-9764.CRC-23-0361)
Supplement: Supplementary Figure 3 — Quality of Life Scores on EQ-5D for ‘usual activities’ over time (other secondary endpoint) (ITT population) [file crc-23-0361-s18.pdf]

Supplementary Figure 3. Quality of Life Scores on EQ-5D for 'usual activities' over time (other secondary endpoint) (ITT population)

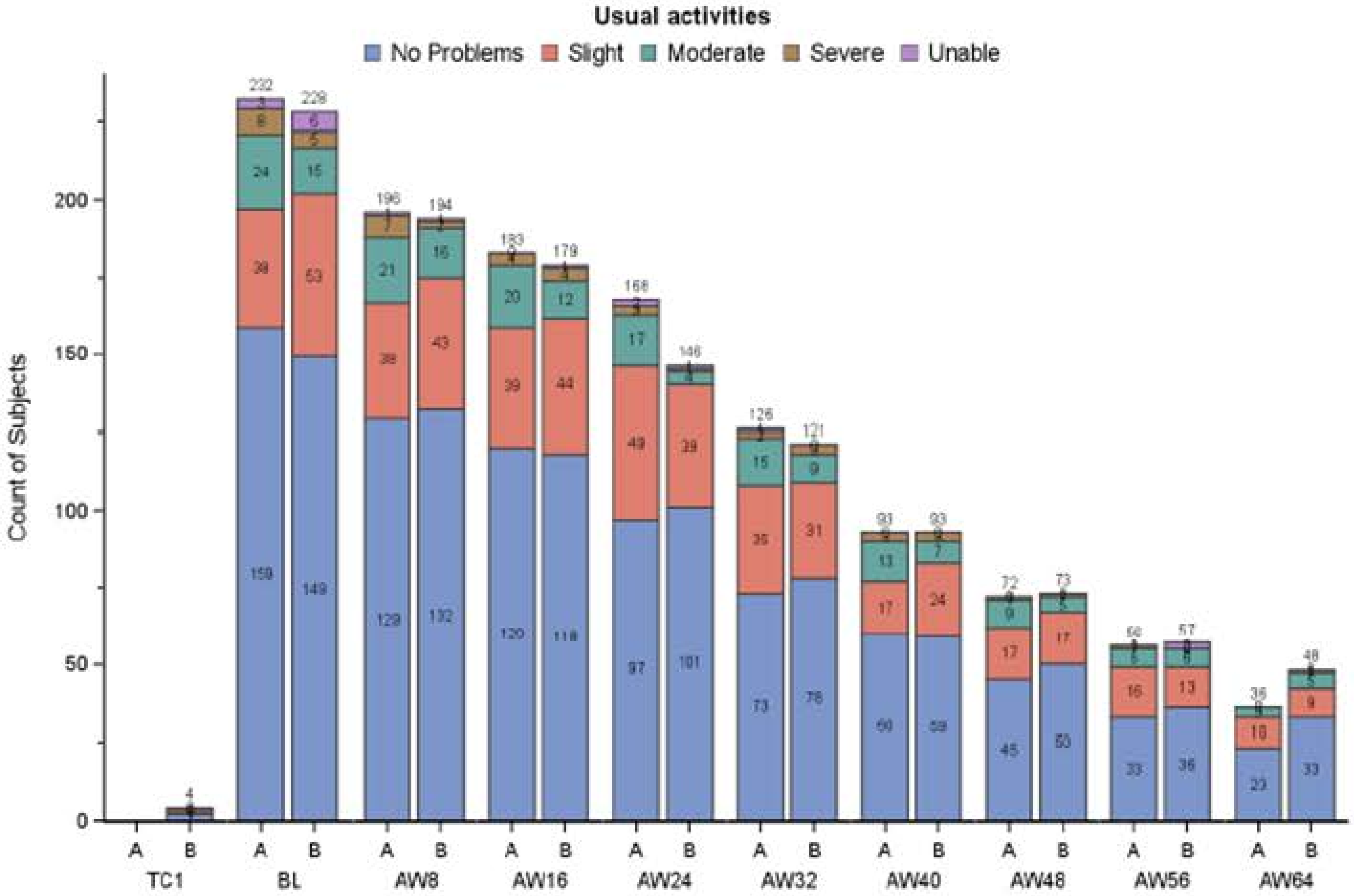

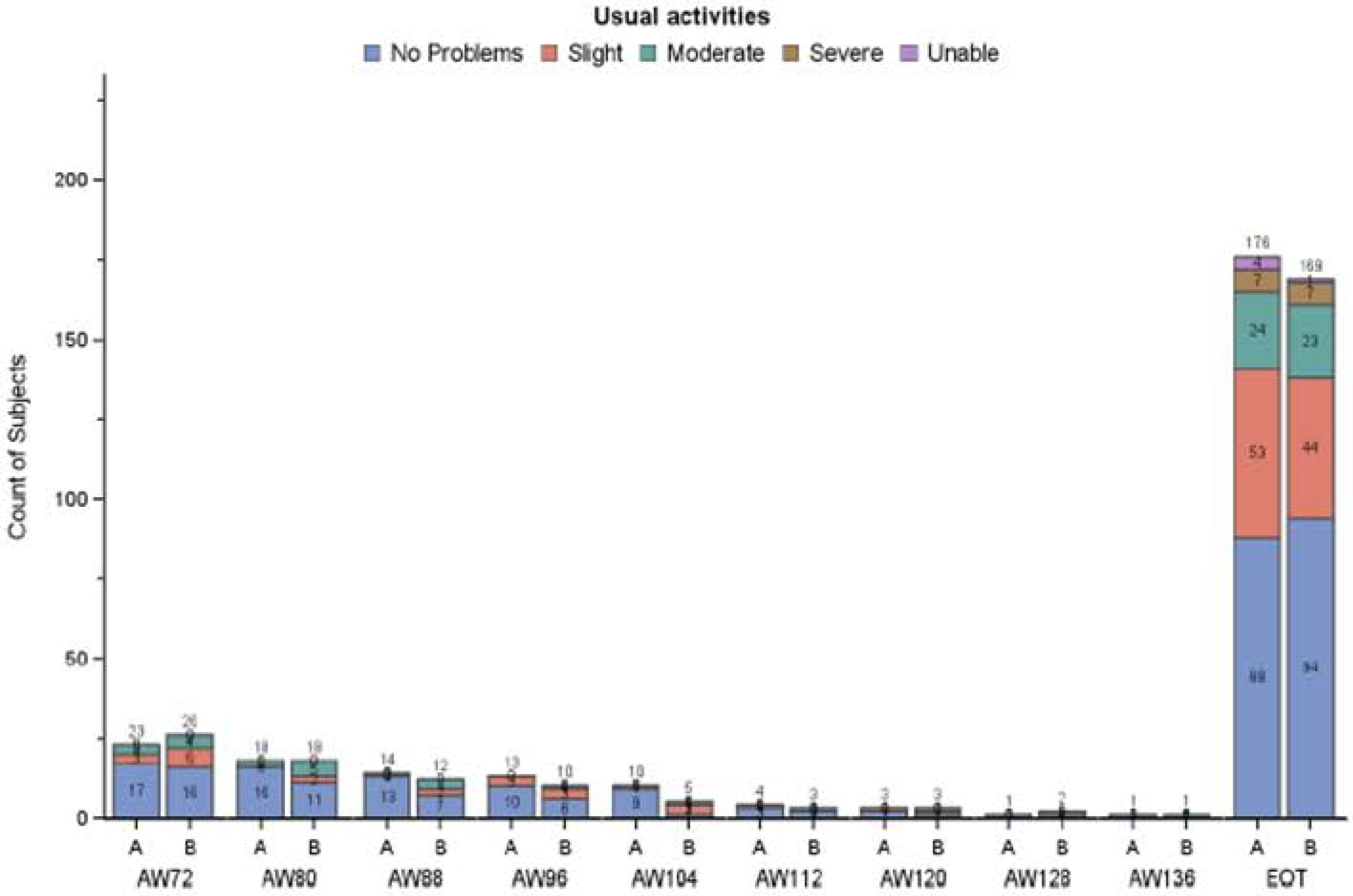

A, arfolitixorin; AW, week of follow-up; B, leucovorin; BL, baseline; EOT, end of treatment; EQ-5D, EuroQol 5 dimensions; ITT, intention-to-treat; TC1, first treatment centre (pre-baseline) visit.
