# Supplementary material for: A Randomized Phase III Study of Arfolitixorin versus Leucovorin with 5-Fluorouracil, Oxaliplatin, and Bevacizumab for First-Line Treatment of Metastatic Colorectal Cancer: The AGENT Trial
Source: Cancer Res Commun. 2024 Jan 4;4(1):28–37. doi: 10.1158/2767-9764.CRC-23-0361 (PMC10765772; doi:10.1158/2767-9764.CRC-23-0361)
Supplement: Supplementary Figure 5 — Association between BRAF Expression and Progression-Free Survival (biomarker analysis set) [file crc-23-0361-s20.pdf]

Supplementary Figure 5. Association between *BRAF* Expression and Progression-Free Survival (biomarker analysis set)

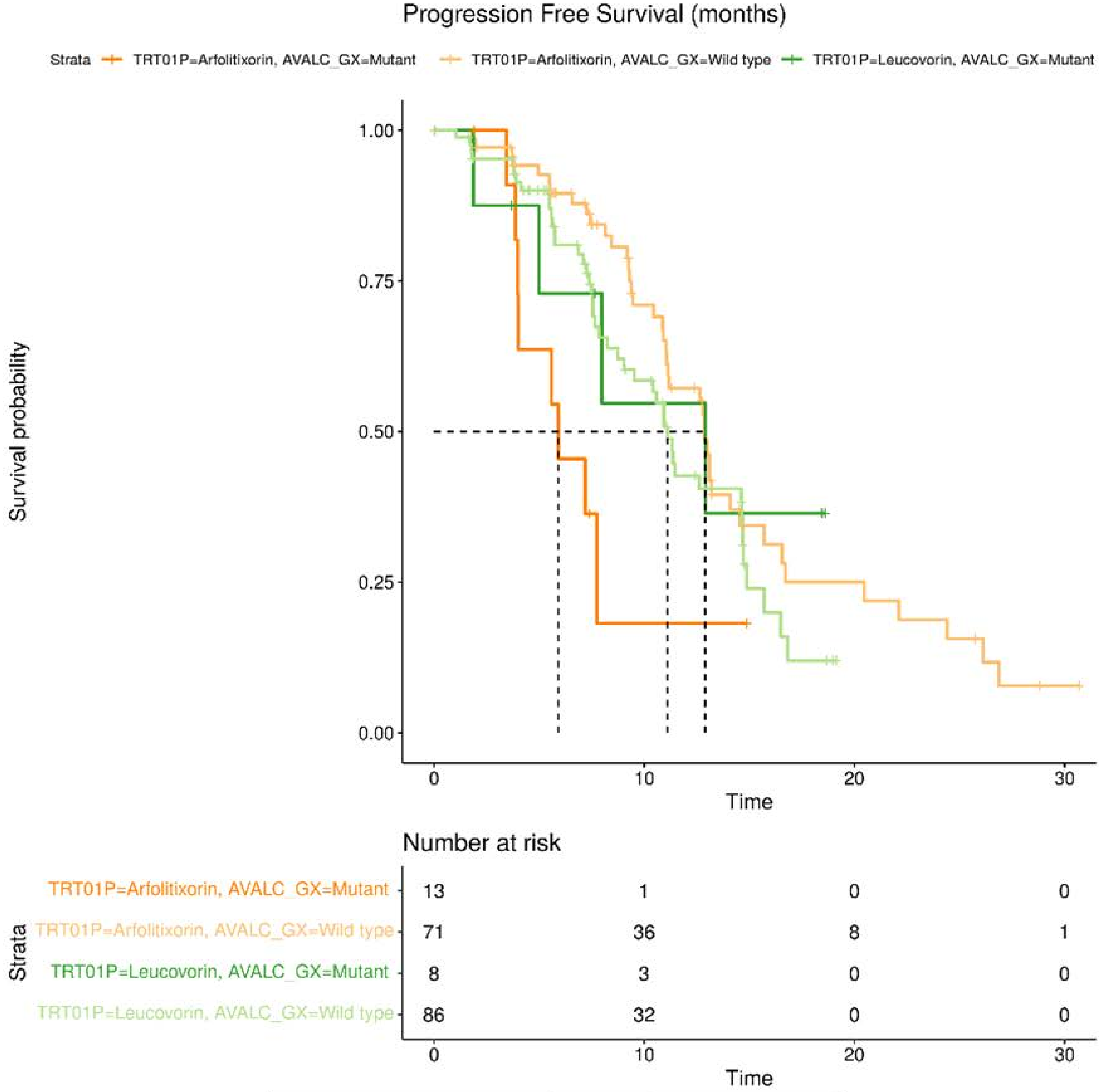

Analysis of Deviance: Term	Statistic	P-value
TRT01P	0.3288	0.5664
AVALC_GX	1.832	0.1759
TRT01P:AVALC_GX	5.705	0.01692

TRT01P, treatment.
