# Supplementary material for: A Randomized Phase III Study of Arfolitixorin versus Leucovorin with 5-Fluorouracil, Oxaliplatin, and Bevacizumab for First-Line Treatment of Metastatic Colorectal Cancer: The AGENT Trial
Source: Cancer Res Commun. 2024 Jan 4;4(1):28–37. doi: 10.1158/2767-9764.CRC-23-0361 (PMC10765772; doi:10.1158/2767-9764.CRC-23-0361)
Supplement: Supplementary Figure 6 — Association between ERCC1 Expression and Progression-Free Survival (biomarker analysis set) [file crc-23-0361-s21.pdf]

Supplementary Figure 6. Association between *ERCC1* Expression and Progression-Free Survival (biomarker analysis set)

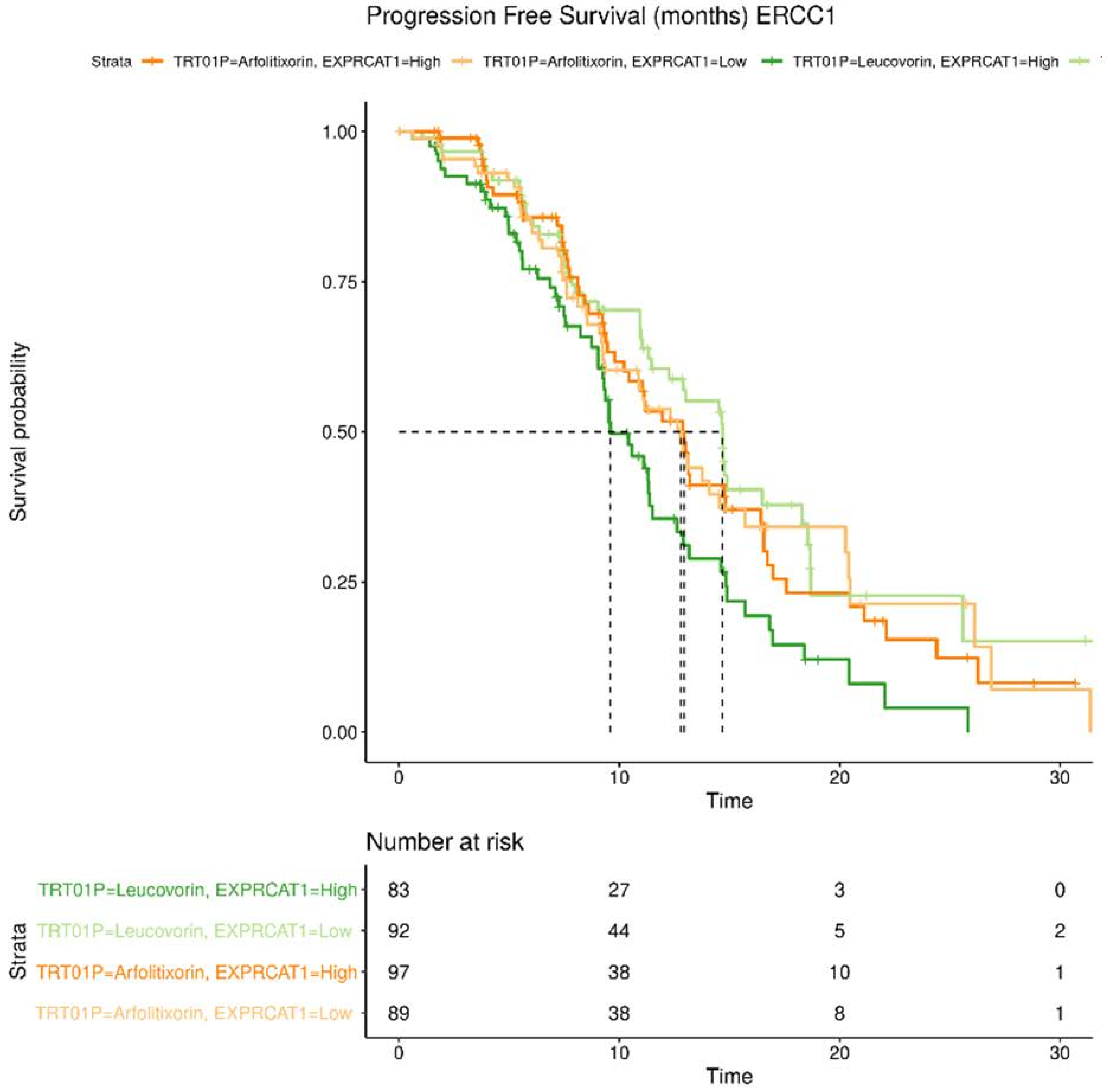

EXPRCAT1, expression; TRT01P, treatment.
